# From periphery immunity to central domain through clinical interview as a new insight on schizophrenia

**DOI:** 10.1038/s41598-024-56344-3

**Published:** 2024-03-08

**Authors:** Wirginia Krzyściak, Marta Szwajca, Natalia Śmierciak, Robert Chrzan, Aleksander Turek, Paulina Karcz, Amira Bryll, Maciej Pilecki, Eva Morava, Anna Ligęzka, Tamas Kozicz, Paulina Mazur, Bogna Batko, Anna Skalniak, Tadeusz Popiela

**Affiliations:** 1 Department of Medical Diagnostic, Faculty of Pharmacy, Jagiellonian University Medical College, 30-688 Krakow, Poland; 2 Department of Child and Adolescent Psychiatry, Faculty of Medicine, Jagiellonian University Medical College, 31-501 Krakow, Poland; 3 Department of Radiology, Faculty of Medicine, Jagiellonian University Medical College, 31-503 Krakow, Poland; 4 Department of Electroradiology, Faculty of Health Sciences, Jagiellonian University Medical College, 31-126 Krakow, Poland; 5 Department of Clinical Genomics, Mayo Clinic, Rochester, MN USA; 6 Department of Research Immunology, Mayo Clinic, Arizona, USA; 7 Division of Molecular Biology and Clinical Genetics, Department of Medicine, Jagiellonian University Medical College, Skawińska 8, 31-066 Krakow, Poland

**Keywords:** Biomarkers, Diseases, Medical research, Risk factors

## Abstract

Identifying disease predictors through advanced statistical models enables the discovery of treatment targets for schizophrenia. In this study, a multifaceted clinical and laboratory analysis was conducted, incorporating magnetic resonance spectroscopy with immunology markers, psychiatric scores, and biochemical data, on a cohort of 45 patients diagnosed with schizophrenia and 51 healthy controls. The aim was to delineate predictive markers for diagnosing schizophrenia. A logistic regression model was used, as utilized to analyze the impact of multivariate variables on the prevalence of schizophrenia. Utilization of a stepwise algorithm yielded a final model, optimized using Akaike’s information criterion and a logit link function, which incorporated eight predictors (White Blood Cells, Reactive Lymphocytes, Red Blood Cells, Glucose, Insulin, Beck Depression score, Brain Taurine, Creatine and Phosphocreatine concentration). No single factor can reliably differentiate between healthy patients and those with schizophrenia. Therefore, it is valuable to simultaneously consider the values of multiple factors and classify patients using a multivariate model.

## Introduction

The aetiology of schizophrenia is recognized as being polygenic, reflecting a multifactorial interplay of sociodemographic, immunological, genetic, and environmental factors. This intricate interrelation contributes to the diagnostic ambiguity and challenges in delineating clear-cut boundaries for schizophrenia. Our preceding research, in line with comprehensive biochemical and genetic investigations, has identified several independent risk factors for schizophrenia, notably including inflammatory processes and immune responses as salient elements linked to its pathogenesis^1,2^.

Gene overlaps in depression, schizophrenia, and cognitive functioning with metabolic processes suggest that both early brain development and neurodegenerative processes influence brain changes^3^. Identifying metabolic changes in the brain aids in determining the biological alterations underlying physiological and pathological brain aging processes, continually influenced by genes and environmental factors.

Brain imaging techniques, essential in assessing cerebral alterations, capture metabolic activity, reduced functional dynamics in the medial prefronatal cortex, mPFC and neurodegenerative changes^4–6^. These techniques offer objective insights into biological changes in the brain, crucial for understanding individual variations in brain development, individual patterns of neural network connections depending on the stage of the disease before treatment, cognitive functioning^7,8^, and risks of psychiatric^9^ and neurological disorders^10,11^, including schizophrenia. These insights are vital for identifying disease risk, treatment resistance, and immune resilience^10^.

Immune dysregulation in schizophrenia is evidenced by increased immune markers in acute psychotic states and altered responses after antipsychotic therapy. Elevated inflammatory factors, such as TNFα, IL-6, IL-17, and CRP, are notably associated with the disorder, especially in treatment-resistant cases^12^. Genetic variants in TNFα's promoter region have been identified as significant in schizophrenia^13^. Recent studies have characterized long-known schizophrenia-specific lymphocytes with unique mitochondrial structures, implicating energy metabolism in the aetiology of schizophrenia^14^. The ‘immune-oxidative’ network, involving cellular immune responses, neuroinflammation, mitochondrial dysfunction, and oxidative stress, is proposed as a mechanism in schizophrenia development^14^.

Traditional neurotransmitter imbalances commonly associated with schizophrenia, such as dopamine, serotonin, and glutamate, have also been observed in cases of low-level neuroinflammation, and they may play a pivotal role in triggering and perpetuating schizophrenia symptoms^14^. The role of chronic inflammation in major mental disorders has garnered increased attention, uncovering numerous potential targets for pharmacological intervention. Neuroimaging studies have also confirmed reduced central nervous system volume and increased microglial activation in individuals with schizophrenia^5^.

Radiological approaches using medical imaging technologies such as Magnetic Resonance Imaging (MRI), Magnetic Resonance Spectroscopy (MRS), Positron Emission Tomography (PET), and Electroencephalography (EEG) offer insights into structural and functional brain anomalies in psychiatric disorders like schizophrenia^4,5^. These techniques aid in revealing phenotypic variables and underlying molecular mechanisms, enhancing our understanding of schizophrenia's polygenic nature^11^. MRI, with advanced modalities like BOLD contrast, fMRI, DTI, and 3D structural imaging, allows detailed analysis of brain tissue composition and subtle changes in psychiatric conditions^15,16^.

MR spectroscopy, particularly H1MRS, is vital for investigating brain metabolism, revealing age and region-dependent variations in metabolic profiles, crucial for understanding the pathogenesis of schizophrenia^17,18^. Abnormal energy metabolism in schizophrenia, often linked to mitochondrial dysfunction and redox imbalance, manifests as altered creatine kinase activity and NAD^+^/NADH ratios in the prefrontal cortex^18^. Chronic schizophrenia patients exhibit distinct metabolic patterns, such as elevated lactate levels and reduced pH, compared to those in their first psychotic episode^19^.

In exploring the posterior cingulate cortex (PCC), a metabolically active brain region, we aim to uncover its yet-unknown relationships, particularly its role in cognitive and affective dysfunctions and its interplay with the anterior cingulate cortex's arousal state^14,20,21^. The PCC's involvement in shaping schizophrenia symptoms, including its impact on network controllability and energy utilization efficiency, is increasingly recognized^14,22,23^. This aligns with findings that excessive spontaneous network switching correlates with symptom intensity in schizophrenia's first episode, pointing to potential mechanisms underlying attentional disturbances during acute psychosis^24^.

Increasing evidence suggests that metabolic changes associated with glucose metabolism, mitochondrial dysfunction, and redox imbalance play a crucial role in the pathophysiology of schizophrenia^18^. In our ongoing quest to unravel the complexities of schizophrenia, our current research builds upon previous studies on energy metabolism in schizophrenia and the differentiation between affected individuals and healthy controls^11,19,25–27^. This study aims to identify patterns of metabolic factors in the central and peripheral domains of brain metabolism and how these factors influence metabolic changes in schizophrenia. We achieve this by integrating clinical assessments with the measurement and comparison of metabolite concentrations in selected brain regions using magnetic resonance spectroscopy. These measurements are further correlated with biochemical parameters, including immunological markers from peripheral blood, and comprehensive clinical evaluations conducted by psychiatrists and psychologists.

A significant goal of our study is to identify commonalities and predictors that distinctly separate individuals with schizophrenia from healthy controls. Unlike many existing studies that lack methodological power and precision, our approach combines and correlates variables from different scales, yielding promising results with an AUC above 0.9, indicating the need for extensive further research in this domain.

The traditional diagnosis of schizophrenia, predominantly reliant on patient-reported symptoms and psychiatrist observations, faces challenges such as varied treatment responses and difficulties in tracking symptom progression. These challenges underscore the imperative need for objective, quantifiable biomarkers that can unravel the underlying pathophysiological mechanisms at molecular, cellular, microcircuitry, and systemic levels. Such an understanding is pivotal for the development of targeted treatment strategies in psychiatric disorders.

Our comprehensive studies on energy metabolism in schizophrenia have shed light on the disorder's complex nature, characterized by a spectrum of structural, functional, and metabolic changes in the brain. These insights not only provide a detailed understanding of schizophrenia's neuropathology but also highlight the necessity for ongoing research. This research is particularly crucial in diagnosing and treating disorders related to mitochondrial dysfunction and exploring their potential for therapeutic interventions.

However, the challenge lies in the fact that these insights, though valuable, have been identified independently and lack the predictive power needed for effective schizophrenia patient management. To address this gap, our current work aims to integrate results from imaging and clinical laboratory tests with patient-reported symptom assessments. This integration allows us to evaluate the influence of various variables on the occurrence of schizophrenia, providing insights into the predictive capacity of our model.

Previous neuroimaging studies have been instrumental in confirming neurochemical changes, especially in the prefrontal cortex and anterior cingulate gyrus of patients with predominantly negative symptoms of schizophrenia^11^. However, these findings have not achieved the specificity required for diagnostic significance. There is an increasing need to move away from ineffective treatments, particularly for patients who do not adequately respond to standard pharmacotherapy. In these scenarios, reliance solely on interview-based diagnoses or individual schizophrenia-associated parameters is insufficient. Thus, our study pioneers the formulation of predictive approaches that amalgamate genetic, biochemical, imaging, and clinical parameters. Integrating these diverse data through advanced statistical models paves the way for a more comprehensive assessment of the diagnostic and predictive potential of selected indicators in schizophrenia. Our study represents the first of its kind to integrate multifaceted factors into a predictive model for schizophrenia, setting a precedent for future research in this field.

## Results

### Characteristics of the sample

The results from *N* = 96 subjects in the groups of healthy subjects (control) and patients with schizophrenia (test) were examined. The study encompassed the outcomes of sociodemographic data, laboratory parameters, clinical assessment parameters, and Magnetic Resonance Spectroscopy (MRS) parameters in two locations (anterior cingulate cortex and posterior cingulate cortex) for two Time to Echo (TE) values (front TE = 30 ms, front TE = 144 ms, rear TE = 30 ms, rear TE = 144 ms).

The comprehensive characteristics of the sociodemographic data for the study sample are presented in Supplementary Table S1, which indicates that there were no significant differences between the groups concerning gender and age.

### Laboratory parameters

The distribution of laboratory parameters for the entire sample and by groups is reported in Supplementary Table S2. According to those findings, there were significant differences between the groups on 22 parameters. In the schizophrenia group, the following parameters were altered.

Significantly elevated:

- White blood cells (WBC) [× 10^3^/µL]
- Neutrophils (NEUT) [× 10^3^/µL]
- Reactive lymphocytes (Re-Lymph) [× 10^3^/µL]
- Immature granulocytes (IG) [× 10^3^/µL]
- Neutrophils (NEUT) [%]
- Reactive lymphocytes (Re-Lymph) [%]
- Red blood cells (RBC) [× 10^6^/µL]
- Hemoglobin (Hgb) [g/dL]
- Hematocrit (Hct) [%]
- Macrocytes [%]
- Potassium (K) [mmol/L]
- Glucose [mmol/L]
- Uric acid [µmol/L]
- Triglycerides [µmol/L]
- Sulfated form of dehydroepiandrosterone (DHEA-S) [µmol/L]
- Insulin [µU/mL]
- Homeostasis model assessment of insulin resistance (HOMA-IR)

Significantly lowered:

- Lymphocytes (lymph) [%]
- Eosinophils (EO) [%]
- Basophils (BASO) [%]
- High-density lipoprotein cholesterol (HDL) [µmol/L]
- Free thyroxine (FT4) [pmol/L]

### Clinical evaluation

The distribution of clinical evaluation scales for all participants in the entire sample and by groups is presented in Table 1 and Supplementary Table S3.

**Table 1:** Baseline clinical characteristics of participants.

Characteristic	Overall N = 96^a^	Group		*p*^b^
		Control, n = 45^a^	Test, n = 51^a^	
GAF score	71.00 (51.75, 100.00)	100.00 (90.00, 100.00)	54.00 (37.50, 63.00)	< 0.001
Missing values	2	2	0	
BDI II score	9.00 (3.00, 20.00)	6.50 (2.00, 13.00)	13.00 (5.00, 28.50)	0.019
Missing values	3	3	0	
STAI score	86.00 (71.50, 103.00)	77.00 (67.50, 90.00)	95.00 (80.00, 110.50)	0.001
Missing values	9	5	4	
GRSR score	21.50 (13.00, 33.00)	25.50 (21.00, 38.50)	17.50 (7.25, 24.75)	< 0.001
Missing values	8	3	5	
GQH 28 score	26.00 (17.00, 39.00)	21.00 (15.00, 28.00)	28.50 (19.25, 42.75)	0.020
Missing values	19	14	5	
TEC score	12.00 (7.00, 24.00)	11.50 (5.00, 17.25)	18.00 (10.00, 33.00)	0.017
Missing values	15	5	10	
ECR-RS score	44.50 (34.00, 56.00)	39.50 (30.00, 47.00)	54.00 (39.50, 62.75)	0.002
Missing values	14	5	9	

^a^Mdn (Q1, Q3).

^b^Wilcoxon rank sum test.

*This information is based on data collected from 96 patients, comprising 45 healthy volunteers and 51 individuals diagnosed with schizophrenia, who underwent clinical examinations and various procedures at the University Hospital in Kraków, Poland. The assessment tools used included GAF (Global Assessment of Functioning), BDI-II (the Beck Depression Inventory), STAI (the State and Trait Anxiety Inventory), GRSR (Gastrointestinal Symptom Rating Scale), GQH 28 (General Health Questionnaire-28), TEC (the Traumatic Experiences Checklist), ECR-RS (the Experiences in Close Relationships-Revised Short). Additionally, the terms and abbreviations used in the presentation are defined as follows: Q1 (first quartile), Q3 (third quartile), *Md* (median), *Q1* (first quartile, 25%), *Q3* (third quartile, 75%).

Our results reveal significant differences between the groups for all seven evaluated parameters, where the schizophrenia group was characterized as follows.

Significantly higher:

- BDI-II (the Beck Depression Inventory) scores
- STAI (the State and Trait Anxiety Inventory) scores
- GQH 28 (General Health Questionnaire-28) scores
- TEC PL (the Traumatic Experiences Checklist) scores
- ECR-RS (the Experiences in Close Relationships-Revised Short) scores

Significantly lower:

- GAF (Global Assessment of Functioning) scores
- Gastric symptoms scores

The medians of positive and negative symptoms were distributed almost equally among the test group (Supplementary Table S3).

### Brain metabolites at two echo times (TE; Front TE = 30 ms, Front TE = 144 ms, Rear TE = 30 ms, Rear TE = 144 ms) in two locations (anterior cingulate cortex, posterior cingulate cortex)

Results of brain imaging analyses are provided in the supplementary data mentioned in each section below, in which they are presented both for all participants in the entire sample as well as detailed by groups.

#### Anterior cingulate cortex (ACC) in TE 30 ms and in TE 144 ms

The distribution of metabolite values in Anterior cingulate cortex at TE 30 ms for all participants in the entire sample and by groups is presented in Supplementary Table S4.

We identified significant differences between the groups for 10 brain parameters, with the following regularities for the schizophrenia group.

Significantly elevated:

- The ratio of Glucose to the sum of creatine and phosphocreatine (Cr + PCr)

Significantly lower:

- Creatine concentration (Creatine conc.)
- Glutamine concentration (Glutamine conc.)
- The ratio of glutamine concentration to the sum of creatine and phosphocreatine concentrations (Glutamine/(Cr + PCr))
- Glutamate concentration (Glutamate conc.)
- Inositol concentration (Inositol conc.)
- *N*-Acetyl aspartate concentration (*N*-acetyl aspartate conc.)
- THE sum of creatine and phosphocreatine concentrations (Cr + PCr conc.)
- The sum of glutamate and glutamine concentrations (Glu + Gln conc.)
- The ratio of the sum of glutamate and glutamine concentrations to the sum of creatine and phosphocreatine concentrations ((Glu + Gln)/(Cr + PCr))

The distribution of metabolite values in the Anterior cingulate cortex at TE 144 ms is presented in Supplementary Table S5 and indicates significant differences between the groups for 10 brain parameters. All those 10 parameters were significantly higher in the control group:

- Phosphocreatine concentration (PCr conc.)
- Glutamate (Glu) concentration
- The ratio of Glutamate to the sum of Creatine and Phosphocreatine concentrations
- *N*-Acetylaspartate concentration (*N*-Acetylaspartate conc.)
- The ratio of *N*-Acetylaspartate to the sum of Creatine and Phosphocreatine concentrations (*N*-Acetylaspartate/(Cr + PCr))
- The sum of *N*-Acetylaspartate and *N*-Acetylspartylglutamate conc.
- The ratio of *N*-Acetylaspartate and *N*-Acetylspartylglutamate to the sum of Creatine and Phosphocreatine concentrations
- The sum of Creatine and Phosphocreatine concentrations (Creatine + Phosphocreatine conc)
- The sum of Glutamate and Glutamine concentrations (Glu + Gln conc.)
- The ratio of the sum of Glutamate and Glutamine concentrations to the sum of Creatine and Phosphocreatine concentrations ((Glu + Gln)/(Cr + PCr))

#### Posterior cingulate cortex (PCC) in TE 30 ms and in TE 144 ms

In the case of the distribution of metabolite values in the Posterior cingulate cortex at TE 30 ms (presented in Supplementary Table S4),the schizophrenia group differed significantly from the control group as follows.

Significantly higher values:

- Taurine concentration
- The ratio of Taurine to the sum of creatine and phosphocreatine (Cr + PCr)

Significantly lower levels:

- Phosphocreatine concentration
- *N*-Acetyl aspartate concentration
- The sum of creatine and phosphocreatine (Cr + PCr) concentration
- Lip20 concentration
- The ratio of Lip20 to the sum of creatine and phosphocreatine (Cr + PCr)

The results presented in Supplementary Table S5 present the distribution of metabolite values in the Posterior cingulate cortex at TE 144 ms and indicate significant differences between the groups across seven brain parameters.

The control group exhibited notably higher levels in:

- L-alanine concentration
- Phosphocreatine concentration
- The ratio of Phosphocreatine concentration to the sum of Creatine and Phosphocreatine concentrations (Phosphocreatine/(Cr + PCr))
- Scylloinositol concentration
- The ratio of Scylloinositol to the sum of Creatine and Phosphocreatine (Scylloinositol / (Cr + PCr)).

Conversely, the control group showed significantly lower levels in:

- The ratio (Glycerophosphocholine + Phosphocholine)/(Cr + PCr)
- The ratio of Creatine/(Cr + PCr).

### Estimating the effects of multiple variables on the schizophrenia occurrence factor

#### Determination of the original regression model

Out of the 63 variables displaying significant univariate effects, 27 potential predictive candidate variables were chosen for inclusion in the primary model, based on literature data and own earlier studies, to elucidate the variation in the occurrence of schizophrenia. These selected variables are as follows: White Blood Cells (WBC [µL]), Neutrophils (NEUT [µL]), reactive lymphocytes (Re-Lymph [µL]), Neutrophils [%], reactive lymphocytes (Re-lymph [%]), red blood cells (RBC [µL]), Glucose [mmol/L], high-density lipoprotein cholesterol (HDL [mmol/L]), Sulfated form of Dehydroepiandrosterone (DHEA-S [µmol/L]), Insulin [µU/mL], Global Assessment of Functioning (GAF score), the Beck Depression Inventory (BDI II score), Glutamine concentration (front rim turn TE 30 ms), Glutamate concentration (front rim turn TE 30 ms), Sum of Glu and Gln concentration (front rim turn TE 30 ms), Glutamate concentration (front rim turn TE 144 ms), N-Acetylaspartate concentration (front rim turn TE 144 ms), N-Acetylaspartate + N-Acetylaspartylglutamate (front rim turn TE 144 ms), Glu + Gln concentration (front rim turn TE 144 ms), Glu + Gln / (Cr + PCr) (front rim turn TE 144 ms), N-acetylaspartate concentration (rear rim turn TE 30 ms), Taurine/(Cr + PCr) (rear rim turn TE 30 ms), Cr + PCr concentration (rear rim turn TE 30 ms), Lip20 concentration (rear rim turn TE 30 ms), L-alanine concentration (rear rim turn TE 144 ms), Scylloinositol concentration (rear rim turn TE 144 ms), and Scylloinositol/(Cr + PCr) (rear rim turn TE 144 ms).

In addition, possible confounding factors have been included in an additional modelling in order to verify their significance. Those variables included age, and sex. It turned out that those factors did not influence the final model.

#### Application of the stepwise algorithm

The fit of the original model was separately evaluated for both the logit and probit link functions using the AIC (Akaike Information Criterion) and BIC (Bayesian Information Criterion) criteria. The AIC metric yielded the smallest criterion value for the logit link function, mainly due to its greater sensitivity to outliers when compared to a logistic sigmoidal curve.

Utilizing a stepwise algorithmic approach, the predictive model underwent refinement, where the initial set of 27 candidate variables was systematically evaluated and pruned down to the eight most predictive variables. This selection process was adjusted for patient age and sex to ensure that the resulting model accounted for these fundamental confounding factors. The effectiveness of the final model refinement was evidenced by a reduction in the AIC value, from an initial 76.5 down to 69.8.

#### Results of fitting the final model

As a consequence of the stepwise algorithm, the ultimate model, fitted through AIC using a logit linking function, encompassed eight predictors and was characterized by the following Eq. (1):

1 $$\begin{gathered} \ln \left( {\frac{{p_{i} }}{{1 - p_{i} }}} \right) = \beta_{0} + \beta_{1} \cdot WBC + \beta_{2} \cdot {\text{Re}} - lymph + + \beta_{3} \cdot RBC \hfill \\ \quad \quad \quad \quad \quad \quad \quad + \beta_{4} \cdot Glu\cos e + \beta_{5} \cdot Insulin ++ \beta_{6} \cdot BDI\,II score + \beta_{7} \cdot Taurine / (Cr + PCr)^{1} \hfill \\ \quad \quad \quad \quad \quad \quad \quad + \beta_{8} \cdot \left( {Cr + PCr} \right)conc^{1} + \beta_{9} \cdot sex + \beta_{10} \cdot age \hfill \\ \end{gathered}$$

^1^Rear rim turn TE 30 ms.

The model exhibited a substantial explanatory power with an *R*^2^_Tjur_ value of 0.67. The intercept of the model, represented as log *OR* and corresponding to female patient with age = 0 years, WBC = 0 µL, Re-lymph = 0 µL, RBC = 0 µL, Glucose = 0 mmol/L, Insulin = 0 µU/mL, BDI II = 0 score, Taurine/(Cr + PCr), and (Cr + PCr) conc = 0 × 10^−6^, was *β*_0_ = 0.00, 95% CI [0.00, 94.90], and *p* = 0.147.

The outcomes of the model that was fitted are displayed in Table 2.

**Table 2:** Results of the fitted logistic regression model, *N* = 96.

Predictors	Occurrence of schizophrenia		
	OR	CI 95%	*p*
Sex [male]	0.33	0.02–3.49	0.372
age	0.91	0.82–1.00	0.068
WBC	1.40	0.85–2.46	0.202
Re-lymph	3.74 × 10^20^	8.91 × 10^7^–6.65 × 10^36^	0.004
RBC	7.77	0.56–158.61	0.143
Glucose	25.02	2.52–507.64	0.014
Insulin	1.10	0.97–1.32	0.227
BDI II score	1.11	1.04–1.22	0.007
Taurine/(Cr + PCr)^a^	1.03	1.01–1.06	0.013
(Cr + PCr) conc^a^	0.90	0.83–0.96	0.008

^a^Rear rim turn TE 30 ms.

WBC, white blood cells; Re-Lymph, reactive lymphocytes; RBC, red blood cells; BDI II, Beck Depression Inventory; Taurine concentration to sum of creatine and phosphocreatine; ratio of Taurine to sum of creatine to phosphocreatine Cr + PCr, sum of concentrations of creatine and phosphocreatine; OR, odds ratios, 95% CI, 95% confidence level (including *ll*—lower limit, *ul*—upper limit); *p*—the *p* value of the statistical test.

The logistic regression model, with a robust Tjur's R-squared value of 0.670, elucidated several parameters with potential etiological relevance to schizophrenia. The model delineated both confounders and predictors, providing a multifaceted view of schizophrenia's correlates.

The model's parameters collectively underscored the relevance of immune-inflammatory processes, metabolic dysregulation, neurotransmitter imbalances, and affective symptoms in the context of schizophrenia.

Sex, a demographic confounder, did not exhibit a significant association with the schizophrenia occurrence in this model, with males showing a reduced, but statistically insignificant, odds ratio (*OR* = 0.33, *p* = 0.372). This finding did not align with the widely recognized modest male predominance in schizophrenia incidence, suggesting that in this sample, sex may not be a determinant factor.

Age revealed an inverse relationship with the likelihood of schizophrenia, with the *OR* of 0.91 indicating a 9% decrement in odds per year increase, approaching significance (*p* = 0.068). This trend underscored the heightened vulnerability among the younger population, resonating with the typical age of onset for schizophrenia during late adolescence to early adulthood.

White blood cell (WBC) count, a proxy for inflammatory status, showed a non-significant elevation in odds (*OR* = 1.40, *p* = 0.202), a finding that was consistent with the hypothesis of a neuroinflammatory component in schizophrenia pathophysiology but lacked the statistical power to corroborate this role.

The parameter of reactive lymphocytes (Re-lymph) was markedly elevated (*OR* = 3.74 × 10^20^, *p* = 0.004), suggesting a profound association with schizophrenia. However, the magnitude of the OR and the breadth of the confidence interval. The significant association between reactive lymphocytes and schizophrenia occurrence may reflect the role of immune system dysregulation in the pathogenesis of the disorder. Some hypotheses suggested that inflammatory processes may contribute to the development of schizophrenia, potentially through the disruption of neurodevelopmental processes or by altering neurotransmitter systems central to the disorder.

Red blood cell (RBC) count was positively associated with schizophrenia occurrence (*OR* = 7.77, *p* = 0.143), though this was not statistically significant within this sample. While the link between RBCs and schizophrenia was not well-established, this could hint at peripheral biomarkers reflecting central nervous system (CNS) pathology. The association between RBC count and schizophrenia, could suggest a link to oxygen transport and by extension, brain metabolism. Abnormalities in RBC indices have been studied for their potential role in various psychiatric conditions, possibly reflecting broader physiological or developmental disturbances.

Glucose levels were significantly associated with schizophrenia (*OR* = 25.02, *p* = 0.014), aligning with the literature on glucose dysregulation in schizophrenia, possibly linked to insulin resistance or disrupted glucoregulatory mechanisms. The strong association between elevated glucose levels and schizophrenia occurrence aligned with previous research indicating that metabolic syndrome was more prevalent among individuals with schizophrenia. This could be related to the disease itself, lifestyle factors, or the side effects of antipsychotic medications, many of which can induce glucose intolerance and weight gain.

Insulin levels, while displaying an *OR* of 1.10 (*p* = 0.227), did not reach statistical significance, suggesting a less pronounced role in schizophrenia within this cohort, despite the established connection between antipsychotic treatment and insulin resistance.

The Beck Depression Inventory II (BDI II) score, indicative of depressive symptomatology, emerged as a significant predictor (*OR* = 1.11, *p* = 0.007), reflecting the comorbidity and symptomatic overlap between depression and schizophrenia, with affective dysregulation being a key component of the schizophrenia spectrum.

The ratio of taurine to creatine plus phosphocreatine (Cr + PCr) presented a subtle but significant increase in odds (*OR* = 1.03, *p* = 0.013), suggesting alterations in amino acid neurotransmitter levels and energy metabolism may play a role in schizophrenia pathology.

Finally, the concentration of creatine plus phosphocreatine (Cr + PCr) itself was inversely associated with schizophrenia occurrence (*OR* = 0.90, *p* = 0.008), potentially reflecting disturbances in brain energy homeostasis and cellular energetics within the disorder.

These findings necessitate replication in broader cohorts and warrant longitudinal studies to ascertain causality and the temporal dynamics of these associations.

The fitted model satisfied the assumptions for the logistic regression model concerning the collinearity parameter, normality of the residuals' distributions, and its ability to replicate the observed data.

The lines predicted by the model in the posterior predictive check closely approximated those of the observed data, providing evidence that the model accurately simulated the actual data. According to the data in Supplementary Table S7, the VIF parameters for all predictors were below 3.0, suggesting a low level of collinearity among the explanatory variables.

Ensuring that the assumptions of the regression model were met allowed us to confidently assert that the predictions, confidence intervals, and scientific observations obtained from the fitted model were not misleading or biased.

**Figure 1:**
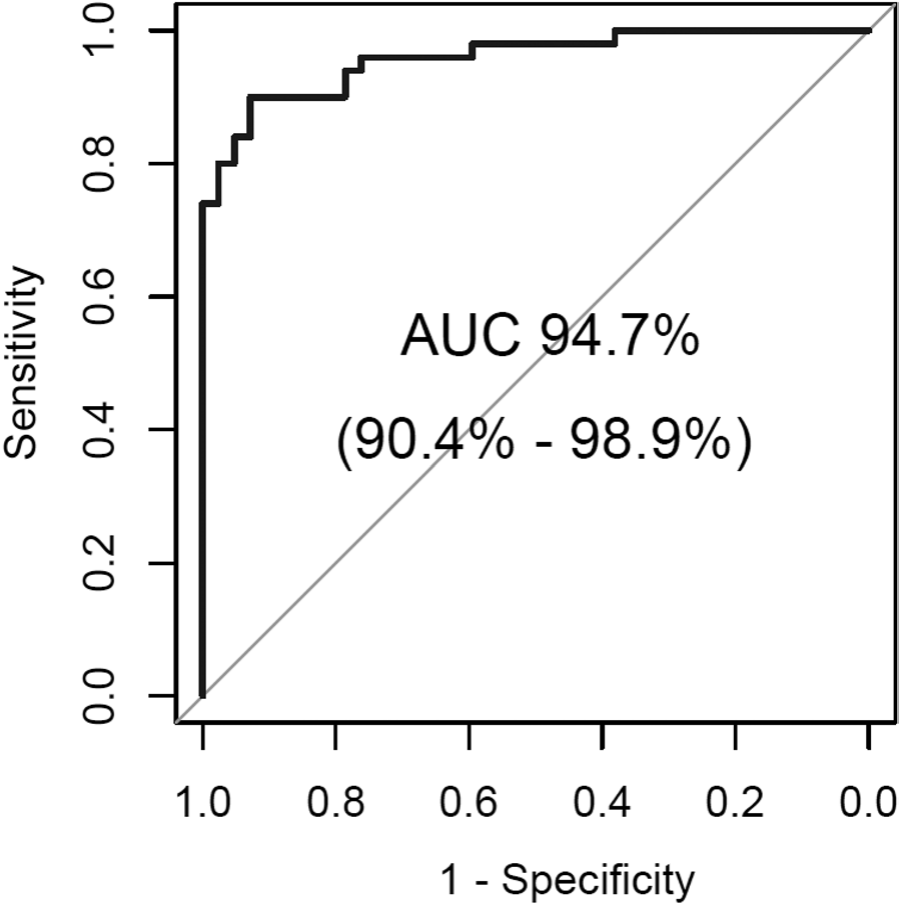
ROC curve with AUC for the fitted final model.

Figure 1 illustrates a model curve of the Receiver Operating Characteristic (ROC) based on the specificity and sensitivity parameters.

The area under the model fit curve was 94.7%, indicating excellent discrimination by the model. The results of the Hosmer–Lemeshow test, *χ*^2^(6) = 1.39, *p* = 0.966, the modified Hosmer–Lemeshow test, *F*(7) = 0.64, *p* = 0.724, and the Osius and Rojek test, *z* < 0.01, *p* = 1.000, all exceeded 5%, suggesting there was no significant difference between the observed data and the predicted values^28,29^. This supports the assumption that the model is a good fit.

The estimation of standardized regression coefficients revealed that reactive lymphocytes (Re-lymph), *β*_std_ = 2.35, CI 95% [0.91, 4.20], and glucose, *β*_std_ = 3.06, CI 95% [0.88, 5.92], had the most significant impact on the schizophrenia incidence factor. In contrast, white blood cells (WBC), *β*_std_ = 0.69, CI 95% [− 0.33, 1.85], red blood cells (RBC), *β*_std_ = 0.92, CI 95% [− 0.26, 2.27], and insulin, *β*_std_ = 0.92, CI 95% [− 0.33, 2.64], had the least effect on the response variable.

### Estimation of marginal effects

The outcomes of the marginal effects estimates for each predictor in the regression model are presented in Supplementary Table S6.

### Estimation of cutoff points for each covariate of the final regression model

The estimated optimal cutpoints with the performance metrics for binary classification for each covariate from the final regression model are presented in Supplementary Table S7 and reveal that the classification metrics for patient groups are fairly average and predominantly consist of a relatively high number of false negatives based on individual predictors.

This led to the conclusion that there are no individual factors that can consistently discriminate between healthy patients and those with schizophrenia. Hence, it is valuable to collectively assess the values of all factors and differentiate patients based on a multivariate model.

## Discussion

Consistent with our earlier findings and the outcomes of other researchers, systemic inflammation and immune mechanisms play a substantial role in the pathogenesis of schizophrenia^2,30^.

While previous research primarily concentrated on measuring cytokines and neurotransmitter metabolites in blood or cerebrospinal fluid, this study adopts a comprehensive approach by considering a broad spectrum of laboratory, cerebral, and clinical parameters. It underscores the complexity of the clinical situation, evidencing that individual parameters may not adequately elucidate or predict outcomes.

Interpreting the eight-factor predictive model developed in this study, the elevated neutrophil and reactive lymphocyte counts may suggest a pathological brain process associated with blood–brain barrier impairment and central nervous system dysregulation, ultimately leading to brain dysfunction. It is also possible that parallel processes with bidirectional effects on the central nervous system and the periphery are being investigated.

Pavlović's study revealed significantly higher total white blood cell counts in individuals with schizophrenia compared to the control group. The literature on this topic suggests a link between the neutrophil-to-lymphocyte ratio (NLR) and elevated leukocyte, neutrophil, and monocyte counts in children and adolescents with schizophrenia^31,32^.

The total white blood cell and neutrophil counts show a positive correlation with glucose levels and a negative correlation with HDL cholesterol levels, as demonstrated by Pavlović et al. in their 2016 study^33^. Our research, akin to the findings of Pavlović et al. and Pillinger et al., reveals elevated levels of glucose and various metabolic parameters in schizophrenia patients, including insulin, the HOMA-IR index, uric acid, and triglycerides when compared to the control group. At the same time, the cardioprotective cholesterol, HDL, is notably lower in the patient group, as indicated by Pillinger et al. in their 2020 study and Pavlović et al. in 2016^33,34^.

The robust positive associations noted in this study between glucose and insulin levels in schizophrenia patients can be independent risk factors for the onset of metabolic syndrome in individuals with schizophrenia^35^.

In a study by Torsvik et al., a positive correlation with triglyceride levels and a negative correlation with "good" HDL cholesterol was prevalent in both schizophrenia and bipolar disorder, resulting in the grouping of patients. Shared transcriptome signatures associated with lipid changes and clinical translational potential were detected, influencing altered innate immunity pathways and resulting in increased expression of the same genes among patients in the shared cluster^36^.

The conducted research revealed a significant increase in scores on the Beck Depression Inventory-II (BDI-II) among individuals with schizophrenia compared to the control group. This finding was particularly intriguing to us, considering our earlier results indicating the presence of phenotypically distinct subgroups of patients with schizophrenia. These subgroups are characterized by variations in glutaminergic transmission as the primary mechanism linking peripheral changes to the brain, ultimately contributing to the emergence of negative symptoms^11^.

Interestingly, there is a notable increase in gastrointestinal symptoms in healthy individuals when compared to those with schizophrenia. This phenomenon may be attributed to an altered perception of pain. Insensitivity to pain in schizophrenia is a multifaceted condition that, in addition to the factors mentioned previously, may also be related to an abundance of negative symptoms. These negative symptoms, prevalent in our group of individuals with schizophrenia, can significantly impact the way patients express their experience of pain^37^. The molecular basis for the observed alterations in pain perception in central nervous system (CNS) disorders like anxiety, schizophrenia, and depression can be attributed to nerve inflammation, a complex process that involves both the peripheral circulation and the central nervous system (CNS)^38^.This concept is reflected in the predictive model of the current study, which combines immunological, metabolic, and central parameters. In the conducted study, apart from the noted metabolic changes in astrocytes that are associated with a reduction in myo-inositol, there are also significant changes in the health and functioning of neurons, which are linked to a notable decrease in the concentration of N-acetyl aspartate when compared to the control group.

This study offers evidence of disruptions in the activation of the hypothalamic–pituitary–adrenal (HPA) axis and abnormal regulatory mechanisms in schizophrenia by revealing increased levels of dehydroepiandrosterone sulfate (DHEA-S) in the blood of individuals with schizophrenia when compared to controls. These findings are in line with research conducted by Babinkostova et al., where patients with schizophrenia exhibited significantly higher levels of cortisol and DHEA-S in their blood in comparison to the control group^39^. DHEA-S, released from the adrenal glands and metabolized in the nervous system, is associated with neurosteroid synthesis and the modulation of brain metabolites, which were notably lower in our patient group in comparison to the controls^40^. Our results are consistent with those of Miodownik et al., who identified elevated levels of DHEA-S, cholesterol, and insulin in individuals with schizophrenia compared to controls^41^.

Our research indicates a diminished presence of energy substances such as creatine and phosphocreatine in the anterior cingulate cortex (ACC) and posterior cingulate cortex (PCC) in individuals with schizophrenia. This reduced concentration of creatine/phosphocreatine is evident at two distinct echo times in both the anterior and posterior cingulate regions, closely associated with the production and utilization of ATP as an energy source in the brains of those affected by schizophrenia. Our observations align with the findings of Sarramea Crespo et al., who observed a decrease in the creatine/phosphocreatine cycle in the cingulate gyrus of patients with comorbid schizophrenia and bipolar affective disorder^42^. PCr/CK plays a critical role in maintaining a stable ATP level during fluctuations in energy demand and connecting ATP production with its utilization site^43^. Various studies have shown that CK activity in the frontal lobe is diminished in patients with chronic schizophrenia, first-episode psychosis (FEP), and the first episode of bipolar affective disorder (BD) with psychotic features^44^. These findings may help elucidate the 8-factor predictive model presented in our research, where the sum of Creatine and Phosphocreatine, Taurine/(Cr + PCr), Beck’s Depression Scale, Glucose (together with Insulin), and Re-Lymph are identified as key predictive markers for schizophrenia.

Currently, the absence of objective biomarkers poses a challenge to diagnostic and therapeutic decisions in schizophrenia. Our study emphasizes the potential diagnostic significance of routine blood parameters (white blood cells, neutrophils, and reactive lymphocytes) and common biochemical markers (thyroxine, glucose, and uric acid) for predicting concurrent metabolic disorders, type 2 diabetes, insulin resistance, as well as thyroid and adrenal issues in individuals with schizophrenia. Furthermore, neuroimaging can provide quantifiable biomarkers that aid in comprehending molecular distinctions in brain circuits.

Highlighting the interplay among the immune system, brain function, peripheral metabolism, and schizophrenia, a comprehensive evaluation involving larger groups of patients has the potential to establish reliable predictors for this intricate disorder in clinical settings. Our research underscores the importance of a personalized, multifaceted model tailored to specific patient subgroups, utilizing quantifiable data from laboratory tests, imaging, clinical investigations, and mental health assessments.

To the best of our knowledge, this is the sole study conducted in humans where the occurrence of schizophrenia was determined by predictive modelling that encompasses laboratory, clinical, and brain-related factors. Supplementary Table S7 provides the estimated optimal cutoff points and the performance metrics for binary classification of each variable derived from the final regression model. The results we obtained confirm our prior findings, which suggested a connection between brain bioenergetic dysfunction and the initial symptoms of schizophrenia. This dysfunction impacts glucose metabolism, insulin resistance, and neuronal development^25^. Disturbances in glutamatergic neurotransmission, nerve inflammation, and redox dysregulation are notable features of individuals with schizophrenia endophenotypes, particularly those exhibiting negative symptoms^11^.

The incorporation of variables such as creatine, phosphocreatine, taurine, neutrophils, and reactive lymphocytes in the ultimate predictive model further underscores the significance of inflammation and oxidative stress in schizophrenia^2^. Alterations in neutrophil and lymphocyte levels signify an underlying pathological brain process resulting in dysfunction, thereby supporting the notion that nerve inflammation is a substantial etiological factor in schizophrenia^45^.

Changes in taurine levels have been observed in individuals with acute polymorphic psychosis and depression, which corresponds with our study where we incorporated the Beck Depression Inventory-II (BDI-II) alongside other predictive factors^46,47^. Wu et al.’s research elucidates the antidepressant impacts of taurine by enhancing the expression of brain-derived neurotrophic factor (BDNF), influencing the survival, proliferation, and differentiation of neural stem cells through the BDNF/ERK/CREB pathway^48^.

The reduced taurine levels in the anterior cingulate cortex (ACC) of individuals with schizophrenia could potentially account for the detected molecular distinctions in glutamine levels, especially in individuals with predominant negative or cognitive symptoms of schizophrenia, as indicated in our earlier investigations^48,49^.

### Limitations

The identified limitations of the study are twofold and are related to the complexity of the data and the size of the participant group.

The study’s data set is inherently complex and multivariate. Although basic demographic variables such as age and gender were included, the small sample size limited the ability to add and analyze a wider range of confounders. Important factors such as type and duration of medication and duration of illness—important for adjusting for confounding effects—were not included in the analysis due to the limited sample.

Furthermore, relying solely on cross-validation within the same sample may not account for all bias specific to a given dataset, potentially biasing the results. This limitation may have an impact on the degree of generalizability of the findings, as there is no data on how well the model will perform in different datasets.

### Summary

Our study’s findings validate the significance of statistical models that rely on a diverse array of numerical variables, highlighting the necessity of integrating neuroimaging parameters, laboratory information, and self-reported assessments in the diagnosis of schizophrenia. Subsequent research efforts should prioritize the examination of molecular mechanisms in individuals displaying a wide range of clinical presentations within the schizophrenia spectrum. The aim is to establish effective treatments for this multifaceted disorder and investigate potential associations between frontal lobe dysfunction, exposure to trauma, stress severity, and comorbid conditions.

## Material and methods

### Participants

Recruitment and clinical assessment were carried out at the Clinical Department of Adult, Child, and Adolescent Psychiatry at the University Hospital in Krakow, Poland. The study enrolled individuals diagnosed with schizophrenia, code F20 according to the ICD-10 criteria confirmed by two independent psychiatrists^10,50^. Furthermore, the severity and symptomatology of the illness were evaluated using the Positive and Negative Syndrome Scale (PANSS)^51^. The schizophrenia affected study participants (N = 51) ranged in age from 13 to 40 years. The participants provided informed consent for the study procedures. For participants under the age of 18, informed consent from a parent or legal guardian was obtained. The project received a favorable opinions from the Jagiellonian University Bioethics Committee: 1072.6120.252.2021 and 1072.6120.178.2022.

The control group comprised 45 healthy volunteers, with an equal gender distribution, aged between 13 and 40 years. These individuals did not have a diagnosis of schizophrenia or any other mental disorders based on the ICD-10 criteria.

The General Health Questionnaire-28 (GHQ-28)^52,53^ was administered to all participants, as it is the most widely used questionnaire for detecting emotional distress and possible psychiatric morbidity in the general population^54^. A comprehensive assessment of current mental, social, and occupational functioning was conducted using the Global Assessment of Functioning (GAF)^55^ based on Axis V in the Diagnostic and Statistical Manual of Mental Disorders, Fourth Edition (DSM-IV-TR)^56^. Gastrointestinal symptoms were evaluated using the Gastrointestinal Symptom Rating Scale (GSRS)^57,58^. All participants completed the Beck Depression Inventory (BDI-II)^59^, the State and Trait Anxiety Inventory (STAI)^60^, and the self-report questionnaire, the Experiences in Close Relationships-Revised Short (ECR-RS), to assess individual attachment style on the two dimensions of anxiety and avoidance experiences in close relationships^61,62^. A retrospective self-assessment of negative and potentially traumatic experiences in childhood and adulthood was also conducted using the Traumatic Experiences Checklist (TEC)^63^, reflecting the total number of potentially traumatic and adverse events over a lifetime. Additional information about participants are available in the Supplementary file.

#### Treatment

In the present study, information from the medical history regarding the use of pharmacological treatments was employed. Medications were administered to schizophrenia patients at therapeutic doses in accordance with the guidelines of the American Psychiatric Association for the treatment of schizophrenia. Patients received these medications in both oral (p.o.) and intramuscular (i.m.) forms, including depot formulations. The patients were prescribed a variety of antipsychotic medications, including aripiprazole, risperidone, perazine, levomepromazine, chlorpromazine, cariprazine, lurasidone, amisulpride, quetiapine, olanzapine, zuclopenthixol, and haloperidol. Notably, one patient was not prescribed any pharmacotherapy. Among the patients, eight were on monotherapy, while the remainder were undergoing polytherapy. Dose conversion of antipsychotics was conducted using chlorpromazine equivalent dose (CPZE), based on the methods described by Davis and Chen, or Andreasen et al., with the exception of amisulpride, for which the daily dose was determined using the WHOCC—ATC/DDD Index, 2023^64–66^. Additionally, lurasidone was administered to seven study participants as per Leucht et al.^67^.

### Blood collection for routine laboratory tests

In the morning, after an 8-h fasting period and overnight rest, on the day of blood collection, prior to medication intake (for chronic medications), blood was collected for comprehensive laboratory examinations from both patients and healthy volunteers. These examinations encompassed a complete blood count, lipid profile (including low-density lipoprotein, high-density lipoprotein, LDL and HDL; triglycerides, TG, and total cholesterol, TC), serum creatinine concentration, alanine aminotransferase (ALT) activity, inflammatory markers (high-sensitivity C-reactive protein, hsCRP), complement components C3 and C4, ionogram (K^+^, Na^+^, Mg^2+^), glucose, insulin, uric acid, HOMA-IR index, as well as thyroid function tests (free triiodothyronine, FT3, free thyroxine, FT4, and thyroid-stimulating hormone, TSH), antibodies against thyroid peroxidase (anti-TPO), adrenal parameter assessment (dehydroepiandrosterone sulfate, DHEA-S), and serum ferritin levels.

Routine analyses were performed in the central laboratory of the University Hospital in Krakow using automated analyzers. The University Hospital laboratory in Krakow undergoes daily internal quality control and systematic external quality control in compliance with established standards for medical diagnostic laboratories.

### Magnetic resonance techniques

Imaging examinations were performed in the MRI unit of the Diagnostic Imaging Department at the Krakow University Hospital, Poland. Additional information about performed Magnetic Resonance Techniques are available in the Supplementary file.

The following metabolites were selected: L-Alanine (Ala 1.48 ppm), Aspartate (Asp 3.8 ppm), Creatine (Cr 3.02 and 3.9 ppm), Phosphocreatine (PCr 3.02 ppm and 3.93 ppm), γ-aminobutyric acid (GABA 2.3 ppm), Glucose (Glc 3.43 and 3.8 ppm), Glutamine (Gln 2.45 and 3.7 ppm), Glutamate (Glu 2.1 and 3.7 ppm), Glycerophosphocholine (GPC 3.6 ppm), Phosphocholine (PCh 4.2 ppm), Glutathione (GSH 3.7 ppm), myo-Inositol (Ins 3.6 ppm), L-Lactate (Lac 1.33 ppm), N-Acetylaspartate (NAA 2.02 ppm), N-Acetylaspartylglutamate (NAAG 4.1 ppm), scyllo-Inositol (Scyllo 3.35 ppm), Taurine (Tau 3.42 ppm), Lipids (Lip09 0.9 ppm, Lip13a and Lip13b 1.3 ppm, Lip20 2.0 ppm), macromolecule (MM09 0.9 ppm, MM12 1.2 ppm, MM14 1.4 ppm, MM17 1.7 ppm, MM20 2.0 ppm). The concentrations of the sums of individual metabolites were also calculated: GPC + PCh, NAA + NAAG, Cr + PCr, Glu + Gln, Lip13a + Lip13b, MM14 + Lip13a + Lip13b + MM12, MM09 + Lip09, MM20 + Lip20. Additionally, the ratios of every metabolite to the sum of creatine and phosphocreatine were calculated, e. g. NAA/(Cr + PCr). Both qualitative and quantitative analysis of the MRS results were performed.

Quality control for the selected model was carried out in relation to the width of the spectral lines and the signal-to-noise ratio.

### Statistical analysis

The manifestation of the schizophrenia factor was assessed using a generalized linear model, as per Eq. (2), leveraging a logit link function for the task.

The logit of the unknown probability of the occurrence of schizophrenia, denoted as *pi*, was modeled as a linear function of the predictors Xi based on equation:

2 $$\log it\left( {p_{i} } \right) = \ln \left( {\frac{{p_{i} }}{{1 - p_{i} }}} \right) = \beta 0 + \beta_{1} \cdot x_{1,i} + \cdots + \beta_{k} \cdot x_{k,i}$$

Additional information about used statistical analysis are available in Supplementary file online.

### Ethical approval

The study received approval from the Jagiellonian University Bioethics Committee under Reference Numbers 1072.6120.252.2021 and 1072.6120.178.2022.

### Supplementary Information

Supplementary Information.

## Data Availability

The datasets generated during and/or analysed during the current study are available from the corresponding author on reasonable request.
