# Supplementary material for: From periphery immunity to central domain through clinical interview as a new insight on schizophrenia
Source: Sci Rep. 2024 Mar 8;14:5755. doi: 10.1038/s41598-024-56344-3 (PMC10923880; doi:10.1038/s41598-024-56344-3)
Supplement: Supplementary file 1 — Supplementary Information. [file 41598_2024_56344_MOESM1_ESM.docx]

**Title:** From Periphery Immunity to Central Domain Through Clinical Interview as a New Insight on Schizophrenia.

**Brief Title:** Statistical Predictive Model of Schizophrenia

Authors: Wirginia Krzyściak^1*^, Marta Szwajca^2^, Natalia Śmierciak^2^, Robert Chrzan^3^, Aleksander Turek^2^, Paulina Karcz^4^, Amira Bryll^3^, Maciej Pilecki^2^, Eva Morava^5^, Anna Ligęzka^6^, Tamas Kozicz^5^, Paulina Mazur^1^, Bogna Batko^2^, Anna Skalniak^7^, Tadeusz Popiela^3^

^1^ Department of Medical Diagnostic, Jagiellonian University Medical College, Faculty of Pharmacy, 30-688 Krakow, Poland

^2^ Department of Child and Adolescent Psychiatry, Jagiellonian University Medical College, Faculty of Medicine, 31-501 Krakow, Poland

^3^ Department of Radiology, Jagiellonian University Medical College, Faculty of Medicine, 31-503 Krakow, Poland

^4^ Department of Electroradiology, Jagiellonian University Medical College, Faculty of Health Sciences, 31-126 Krakow, Poland

^5^ Department of Clinical Genomics, Mayo Clinic, Rochester Minnesota, USA

^6^ Department of Research Immunology, Mayo Clinic, Arizona, USA

^7^ Division of Molecular Biology and Clinical Genetics, Department of Medicine, Jagiellonian University Medical College, Skawińska 8, 31-066, Krakow, Poland

**Corresponding author**:

Wirginia Krzyściak

Department of Medical Diagnostic

Jagiellonian University Medical College, Faculty of Pharmacy

30-688 Kraków, Poland

E-mail: [wirginiakrzysciak@cm-uj.krakow.pl](mailto:wirginiakrzysciak@cm-uj.krakow.pl)

| *Characteristic* | *N* | *Overall* | *Group* | | *p* |
| --- | --- | --- | --- | --- | --- |
|  |  |  | *control, n = 45^1^* | *test, n = 51^1^* |  |
| Sex | 96 |  |  |  | 0.330*^3^* |
| female |  | 44.0 (45.8%) | 23.0 (51.1%) | 21.0 (41.2%) |  |
| male |  | 52.0 (54.2%) | 22.0 (48.9%) | 30.0 (58.8%) |  |
| Age [years] | 96 | 30.5 (22.8, 36.0)*^2^* | 32.0 (27.0, 36.0) *^2^* | 30.0 (18.0, 35.5) *^2^* | 0.100*^4^* |
| ^1^ *n* (%)  ^2^ *Mdn* (*Q1, Q3*) | | | | | |
| ^3^ Pearson's Chi-squared test  ^4^ Wilcoxon rank sum test | | | | | |

Supplementary Table S1. Krzyściak Wirginia, Title: Statistical Predictive Model of Schizophrenia. The distribution of sociodemographic variables overall and by group.

| *Characteristic* | *Overall*  *N = 96^1^* | *Group* | | *p* |
| --- | --- | --- | --- | --- |
|  |  | *control,*  *n = 45^1^* | *test,*  *n = 51^1^* |  |
| WBC [×10^3^/µL] | 6.61 (5.39, 7.46) | 5.77 (4.91, 7.14) | 6.93 (5.73, 8.45) | **0.004** |
| NEUT [×10^3^/µL] | 3.42 (2.69, 4.41) | 2.96 (2.41, 3.72) | 4.10 (3.05, 5.58) | **<0.001** |
| Lymph [×10^3^/µL] | 2.19 (1.66, 2.54) | 2.12 (1.69, 2.50) | 2.24 (1.60, 2.59) | 0.866 |
| Re-Lymph [×10^3^/µL] | 0.05 (0.03, 0.08) | 0.04 (0.03, 0.06) | 0.07 (0.04, 0.10) | **<0.001** |
| As-Lymph_[×10^6^/µL] | 0.00 (0.00, 10.00) | 0.00 (0.00, 10.00) | 0.00 (0.00, 10.00) | 0.228 |
| MONO [×10^3^/µL] | 0.54 (0.47, 0.65) | 0.51 (0.43, 0.63) | 0.56 (0.50, 0.68) | 0.067 |
| EO [×10^3^/µL] | 0.13 (0.09, 0.21) | 0.14 (0.10, 0.21) | 0.12 (0.08, 0.24) | 0.238 |
| BASO [×10^3^/µL] | 0.04 (0.03, 0.05) | 0.04 (0.03, 0.05) | 0.04 (0.03, 0.06) | 0.988 |
| IG [×10^3^/µL] | 0.02 (0.01, 0.02) | 0.01 (0.01, 0.02) | 0.02 (0.01, 0.03) | **0.003** |
| NEUT [%] | 54.80 (48.53, 61.85) | 52.60 (46.10, 55.20) | 58.50 (52.05, 65.40) | **<0.001** |
| Lymph [%] | 33.15 (27.32, 37.95) | 34.80 (32.00, 39.50) | 30.30 (23.25, 37.25) | **0.001** |
| Re-Lymph [%] | 0.80 (0.60, 1.20) | 0.60 (0.40, 0.90) | 0.90 (0.70, 1.20) | **0.003** |
| As-Lymph [%] | 0.00 (0.00, 0.13) | 0.00 (0.00, 0.20) | 0.00 (0.00, 0.10) | 0.570 |
| MONO [%] | 8.50 (6.90, 9.93) | 8.60 (7.20, 10.20) | 8.20 (6.65, 9.60) | 0.220 |
| EO[%] | 2.00 (1.30, 3.12) | 2.40 (1.70, 3.50) | 1.70 (1.10, 2.80) | **0.004** |
| BASO [%] | 0.60 (0.40, 0.83) | 0.70 (0.50, 0.90) | 0.60 (0.40, 0.80) | **0.041** |
| IG [%] | 0.20 (0.00, 0.30) | 0.10 (0.00, 0.30) | 0.20 (0.00, 0.40) | 0.105 |
| NEUT-GI (granulocyte granularity) | 151.60 (149.55, 154.33) | 151.60 (149.10, 154.40) | 151.60 (149.75, 153.85) | 0.915 |
| NEUT-RI (Granulocyte reactivity) | 45.80 (44.55, 47.62) | 45.70 (44.60, 47.70) | 46.00 (44.40, 47.40) | 0.974 |
| RBC [×10^6^/µL] | 4.81 (4.56, 5.07) | 4.69 (4.43, 4.86) | 4.99 (4.68, 5.21) | **0.003** |
| Hgb [g/dL] | 14.40 (13.57, 15.60) | 14.40 (13.50, 14.90) | 14.80 (13.70, 16.35) | **0.037** |
| Hct [%] | 42.00 (39.75, 44.82) | 41.40 (39.20, 42.80) | 43.10 (40.35, 46.35) | **0.012** |
| MCV [fl] | 88.40 (85.65, 90.20) | 88.00 (85.30, 90.20) | 89.00 (85.75, 90.00) | 0.727 |
| MCH [pg] | 30.40 (29.60, 31.00) | 30.50 (29.50, 31.20) | 30.20 (29.60, 30.95) | 0.305 |
| MCHC [g/dL] | 34.25 (33.77, 34.90) | 34.40 (33.90, 35.10) | 34.10 (33.60, 34.70) | 0.082 |
| RDW-SD [fl] | 40.45 (38.90, 42.75) | 40.30 (38.90, 42.40) | 40.60 (39.00, 42.90) | 0.909 |
| RDW-CV [%] | 12.80 (12.30, 13.20) | 12.80 (12.40, 13.10) | 12.70 (12.20, 13.20) | 0.848 |
| Microcytes [%] | 1.50 (1.00, 2.10) | 1.50 (1.00, 2.20) | 1.60 (1.00, 2.10) | 0.721 |
| Macrocytes [%] | 3.90 (3.70, 4.20) | 3.80 (3.60, 4.10) | 4.00 (3.80, 4.30) | **0.041** |
| PLT [×10^3^/µL] | 261.00 (220.00, 302.75) | 275.00 (231.00, 306.00) | 250.00 (213.00, 299.50) | 0.093 |
| PDW [fl] | 12.60 (11.50, 14.05) | 12.30 (11.50, 13.20) | 13.00 (11.53, 14.52) | 0.155 |
| Missing values | 1 | 0 | 1 |  |
| MPV [fl] | 10.70 (10.10, 11.40) | 10.70 (10.30, 11.30) | 10.90 (10.10, 11.60) | 0.531 |
| Missing values | 1 | 0 | 1 |  |
| P-LCR [%] | 31.00 (25.65, 36.00) | 30.10 (26.70, 33.20) | 32.55 (25.52, 38.52) | 0.320 |
| Missing values | 1 | 0 | 1 |  |
| PCT [%] | 0.27 (0.24, 0.33) | 0.29 (0.25, 0.33) | 0.26 (0.22, 0.32) | 0.115 |
| Missing values | 1 | 0 | 1 |  |
| CRP hs [mg/L] | 0.96 (0.36, 1.90) | 0.91 (0.38, 1.29) | 1.11 (0.35, 2.82) | 0.166 |
| Complement, C3c component [g/L] | 1.15 (1.00, 1.28) | 1.11 (1.00, 1.22) | 1.19 (1.03, 1.31) | 0.200 |
| Complement, C4 component [g/L] | 0.21 (0.19, 0.25) | 0.22 (0.20, 0.26) | 0.20 (0.18, 0.25) | 0.235 |
| Na [mmol/L] | 140.00 (139.00, 141.00) | 140.00 (139.00, 141.00) | 140.00 (139.00, 142.00) | 0.087 |
| K [mmol/L] | 4.41 (4.19, 4.67) | 4.30 (4.12, 4.55) | 4.49 (4.30, 4.73) | **0.006** |
| Glucose [mmol/L] | 4.69 (4.38, 5.06) | 4.52 (4.15, 4.88) | 4.82 (4.57, 5.30) | **<0.001** |
| Creatinine [µmol/L] | 76.90 (65.68, 84.65) | 75.50 (68.20, 84.40) | 78.50 (63.95, 85.75) | 0.817 |
| GFR by MDRD [ml/min/1.73m^2^] | 90.00 (89.75, 90.00) | 90.00 (86.00, 90.00) | 90.00 (90.00, 90.00) | 0.154 |
| Uric acid [µmol/L] | 306.00 (246.00, 354.50) | 290.00 (243.00, 334.00) | 320.00 (275.00, 362.50) | **0.049** |
| Cholesterol [µmol/L] | 4.80 (4.30, 5.45) | 5.00 (4.40, 5.50) | 4.65 (4.15, 5.38) | 0.279 |
| Missing values | 1 | 0 | 1 |  |
| Cholesterol HDL [µmol/L] | 1.49 (1.18, 1.78) | 1.60 (1.44, 1.85) | 1.30 (1.10, 1.56) | **<0.001** |
| Missing values | 1 | 0 | 1 |  |
| LDL Cholesterol calculated [µmol/L] | 2.70 (2.10, 3.20) | 2.70 (2.30, 3.20) | 2.70 (2.03, 3.10) | 0.490 |
| Missing values | 1 | 0 | 1 |  |
| Triglycerides [µmol/L] | 1.11 (0.74, 1.70) | 0.98 (0.70, 1.46) | 1.42 (0.79, 1.89) | **0.013** |
| Missing values | 1 | 0 | 1 |  |
| ALT [U/L] | 21.50 (17.00, 35.25) | 20.00 (16.00, 27.00) | 24.00 (17.00, 41.50) | 0.059 |
| TPO [U/mL] | 9.00 (9.00, 9.72) | 9.00 (9.00, 10.30) | 9.00 (9.00, 9.00) | 0.395 |
| Missing values | 1 | 0 | 1 |  |
| TSH [µIU/mL] | 1.97 (1.46, 2.66) | 1.97 (1.61, 2.46) | 1.97 (1.17, 2.78) | 0.559 |
| FT3[pmol/L] | 5.26 (4.70, 5.76) | 5.38 (4.85, 6.10) | 5.21 (4.56, 5.65) | 0.074 |
| FT4 [pmol/L] | 15.75 (14.20, 17.22) | 16.40 (15.00, 17.90) | 15.40 (14.10, 16.60) | **0.027** |
| DHEA-S [µmol/L] | 7.35 (4.49, 9.75) | 6.25 (3.93, 8.37) | 8.64 (5.50, 10.80) | **0.005** |
| Insulin [µU/mL] | 9.96 (6.99, 14.20) | 8.12 (6.29, 10.30) | 12.00 (8.86, 18.50) | **<0.001** |
| HOMA-IR | 2.09 (1.45, 3.04) | 1.56 (1.19, 2.17) | 2.65 (1.90, 4.72) | **<0.001** |
| Missing values | 2 | 0 | 2 |  |
| Ferritin [ug/L] | 74.50 (37.25, 119.50) | 84.00 (35.00, 110.00) | 74.00 (46.00, 123.00) | 0.736 |
| *^1^* *Mdn* (*Q1, Q3*)  *^2^* Wilcoxon rank sum test | | | | |

* Available in 96 patients (45 healthy volunteers and 51 with diagnosed schizophrenia) who underwent clinical examination and other procedures in University Hospital, Kraków, Poland. Complete blood count (CBC) test: WBC, white blood cells; NEUT, neutrophils; Lymph, lymphocytes; Re-Lymph, reactive lymphocytes, parameters of the immune response activation; As-Lymph, activated B lymphocytes (plasma cells) that synthesize antibodies; MONO, monocytes; EO, eosinophils; BASO, basophils; IG, Immature Granulocyte; RBC, red blood cells; Hgb, hemoglobin; Hct, hematocrit; MCV, Mean Corpuscular Volume; MCH, mean corpuscular hemoglobin; MCHC, mean corpuscular hemoglobin concentration; RDW-SD, red cell distribution width - standard deviation; RDW-CV, red cell distribution width - coefficient of variation; PLT, platelet; PDW, Platelet volume distribution width; MPV, Mean platelet volume; P-LCR, Platelet larger cell ratio; PCT, Plateletcrit; CRPhs, The high-sensitivity C-reactive protein; GFR, estimating the glomerular filtration rate (GFR); HDL, high-density lipoprotein cholesterol; LDL, low-density lipoprotein cholesterol; ALT, Alanine aminotransferase; TPO, Thyroid peroxidase antibodies; TSH, Thyroid-stimulating hormone; FT4, free thyroxine; FT3, free tri-iodothyronine; DHEA-S, Sulfated form of Dehydroepiandrosterone; HOMA-IR, homeostasis model assessment of insulin resistance.

Supplementary Table S2. Krzyściak Wirginia, Title: Statistical Predictive Model of Schizophrenia. Baseline results of laboratory tests. Data is presented as median (Q1; Q3); p—the p-value of the statistical test.

| *Characteristic* | *N* | *Distribution^1^* |
| --- | --- | --- |
| *PANSS* | | |
| Positive symptoms | 50 | 22.00 (17.25, 25.75) |
| Negative symptoms | 50 | 23.00 (15.25, 25.75) |
| Disorganized speech | 50 | 17.00 (13.00, 21.00) |
| Uncontrolled hostility excitement | 50 | 8.00 (5.00, 9.00) |
| Anxiety depression | 50 | 12.00 (9.00, 14.00) |
| P1-P7 | 50 | 19.00 (14.00, 22.00) |
| N1-N7 | 50 | 22.00 (15.00, 26.00) |
| G1-G16 | 50 | 39.50 (35.00, 47.00) |
| Total score | 50 | 80.00 (70.00, 93.00) |
| *^1^ Mdn (Q1, Q3)* | | |

Supplementary Table S3. Krzyściak Wirginia, Title: Statistical Predictive Model of Schizophrenia. Severity and symptomatology of the illness were assessed using the Positive and Negative Syndrome Scale (the PANSS). Distributions of questionnaire results for the test group.

|  | *Overall,*  *N = 96^1^* | *ACC*  *Group* | | *p* | *Overall,*  *N = 96^1^* | *PCC*  *Group* | | *p* |
| --- | --- | --- | --- | --- | --- | --- | --- | --- |
|  |  | *control, n = 45^1^* | *test, n = 51^1^* |  |  | *control, n = 45^1^* | *test, n = 51^1^* |  |
| L-alanine conc. [×10^-6^] | 0.00 (0.00, 6.18) | 0.00 (0.00, 8.50) | 0.00 (0.00, 5.56) | 0.733 | 0.00 (0.00, 7.72) | 0.00 (0.00, 8.77) | 0.00 (0.00, 7.66) | 0.712 |
| L-alanine / (Cr+PCr) [×10^-3^] | 0.00 (0.00, 35.00) | 0.00 (0.00, 49.00) | 0.00 (0.00, 33.00) | 0.812 | 0.00 (0.00, 0.06) | 0.00 (0.00, 0.06) | 0.00 (0.00, 0.06) | 0.783 |
| Asparaginate conc. [×10^-6^] | 31.10 (21.00, 41.30) | 32.30 (24.90, 40.85) | 29.75 (19.50, 41.67) | 0.452 | 31.60 (23.40, 41.60) | 31.60 (23.50, 43.00) | 31.65 (23.55, 38.05) | 0.776 |
| Asparaginate / (Cr+PCr) | 0.17 (0.12, 0.22) | 0.17 (0.14, 0.22) | 0.17 (0.12, 0.23) | 0.874 | 0.23 (0.17, 0.28) | 0.22 (0.16, 0.28) | 0.23 (0.18, 0.29) | 0.680 |
| Creatine conc. [×10^-6^] | 86.20 (79.80, 98.60) | 92.10 (83.70, 103.50) | 84.20 (75.40, 92.38) | **0.029** | 77.00 (69.00, 85.70) | 77.50 (70.05, 90.45) | 76.10 (65.58, 82.55) | 0.231 |
| Creatine / (Cr+PCr) | 0.50 (0.45, 0.55) | 0.51 (0.45, 0.55) | 0.49 (0.45, 0.54) | 0.942 | 0.53 (0.48, 0.59) | 0.53 (0.49, 0.59) | 0.55 (0.48, 0.58) | 0.538 |
| Phosphocreatine conc. [×10^-6^] | 88.50 (75.80, 98.10) | 91.40 (78.65, 105.00) | 88.10 (73.90, 96.70) | 0.086 | 0.07 (0.06, 0.08) | 0.07 (0.06, 0.08) | 0.06 (0.05, 0.07) | **0.047** |
| Phosphocreatine / (Cr+PCr) | 0.50 (0.45, 0.55) | 0.49 (0.45, 0.55) | 0.51 (0.46, 0.55) | 0.942 | 0.47 (0.41, 0.52) | 0.47 (0.41, 0.51) | 0.45 (0.42, 0.52) | 0.538 |
| Gamma-aminobutyric acid conc. [×10^-6^] | 35.90 (26.30, 45.00) | 35.60 (25.45, 44.50) | 37.00 (28.95, 45.53) | 0.862 | 19.90 (14.80, 26.60) | 21.50 (16.35, 26.55) | 18.15 (13.00, 26.38) | 0.220 |
| Gamma-aminobutyric acid / (Cr+PCr) | 0.22 (0.15, 0.26) | 0.22 (0.14, 0.25) | 0.23 (0.17, 0.27) | 0.175 | 0.13 (0.11, 0.19) | 0.14 (0.12, 0.17) | 0.13 (0.09, 0.20) | 0.566 |
| Glucose conc. [×10^-6^] | 26.90 (20.10, 35.40) | 25.30 (19.85, 30.65) | 28.95 (20.58, 38.40) | 0.092 | 17.60 (13.10, 25.30) | 16.40 (11.20, 24.25) | 18.40 (14.30, 26.20) | 0.298 |
| Glucose / (Cr+PCr) | 0.15 (0.11, 0.20) | 0.15 (0.11, 0.17) | 0.18 (0.12, 0.23) | **0.014** | 0.13 (0.09, 0.17) | 0.11 (0.08, 0.17) | 0.14 (0.10, 0.18) | 0.091 |
| Glutamine conc. [×10^-6^] | 107.00 (89.70, 126.00) | 119.00 (101.00, 133.50) | 99.60 (82.70, 114.50) | **<0.001** | 91.70 (76.60, 105.00) | 95.00 (79.45, 110.50) | 90.90 (74.03, 103.75) | 0.235 |
| Glutamine / (Cr+PCr) | 0.62 (0.52, 0.72) | 0.65 (0.56, 0.74) | 0.59 (0.49, 0.69) | **0.024** | 0.63 (0.54, 0.71) | 0.62 (0.55, 0.68) | 0.63 (0.54, 0.71) | 0.658 |
| Glutamate conc. [×10^-6^] | 282.00 (261.00, 312.00) | 300.00 (274.00, 331.00) | 268.50 (250.00, 300.75) | **0.001** | 220.00  (197.00, 245.00) | 229.00  (201.00, 248.50) | 216.00  (196.25, 238.25) | 0.143 |
| Glutamate / (Cr+PCr) | 1.64 (1.53, 1.72) | 1.64 (1.57, 1.73) | 1.64 (1.49, 1.71) | 0.289 | 1.54 (1.42, 1.67) | 1.50 (1.35, 1.59) | 1.57 (1.44, 1.67) | 0.096 |
| Glutathione conc. [×10^-6^] | 59.80 (54.00, 66.70) | 60.40 (52.45, 68.25) | 59.50 (55.80, 65.65) | 0.439 | 54.80 (48.00, 61.10) | 56.20 (47.10, 63.05) | 53.95 (48.27, 58.38) | 0.298 |
| Glutathione / (Cr+PCr) | 0.34 (0.31, 0.38) | 0.33 (0.29, 0.38) | 0.35 (0.32, 0.38) | 0.245 | 0.37 (0.33, 0.42) | 0.36 (0.32, 0.41) | 0.38 (0.35, 0.42) | 0.215 |
| myo-Inositol conc. [×10^-6^] | 157.00 (145.00, 171.00) | 164.00 (150.00, 177.00) | 154.00 (145.00, 166.00) | **0.014** | 122.00  (110.00, 132.00) | 123.00  (114.50, 134.00) | 118.00  (107.25, 128.75) | 0.143 |
| myo-Inositol / (Cr+PCr) | 0.90 (0.85, 0.96) | 0.89 (0.84, 0.93) | 0.92 (0.85, 0.98) | 0.181 | 0.83 (0.79, 0.89) | 0.83 (0.75, 0.88) | 0.85 (0.80, 0.90) | 0.162 |
| L-lactate conc. [×10^-6^] | 16.10 (8.38, 24.20) | 18.20 (10.35, 24.15) | 15.85 (7.02, 24.48) | 0.337 | 11.30 (2.92, 24.60) | 10.50 (3.37, 22.85) | 11.95 (2.51, 25.75) | 0.954 |
| L-lactate / (Cr+PCr) | 0.09 (0.05, 0.14) | 0.10 (0.06, 0.13) | 0.09 (0.04, 0.14) | 0.553 | 0.07 (0.02, 0.17) | 0.07 (0.02, 0.16) | 0.08 (0.02, 0.18) | 0.760 |
| N-acetylaspartate conc. [×10^-6^] | 209.00 (196.00, 225.00) | 222.00 (209.50, 230.00) | 199.00 (185.00, 213.25) | **<0.001** | 194.00  (184.00, 205.00) | 199.00  (191.00, 209.50) | 188.00  (180.00, 198.25) | **<0.001** |
| N-acetylaspartate / (Cr+PCr) | 1.19 (1.13, 1.25) | 1.21 (1.15, 1.27) | 1.18 (1.12, 1.24) | 0.213 | 1.34 (1.26, 1.44) | 1.34 (1.24, 1.41) | 1.34 (1.27, 1.46) | 0.282 |
| N-acetylaspartylglutamate conc. [×10^-6^] | 27.40 (21.20, 34.10) | 30.80 (21.65, 35.95) | 25.35 (20.82, 33.52) | 0.100 | 31.00 (23.90, 36.50) | 31.80 (21.40, 37.55) | 30.55 (24.95, 34.88) | 0.616 |
| N-acetylaspartylglutamate / (Cr+PCr) | 0.16 (0.12, 0.19) | 0.17 (0.13, 0.19) | 0.15 (0.12, 0.18) | 0.439 | 0.21 (0.17, 0.25) | 0.22 (0.16, 0.25) | 0.21 (0.18, 0.25) | 0.600 |
| Scyllo-inositol conc. [×10^-6^] | 1.93 (0.00, 3.80) | 2.49 (0.41, 3.79) | 0.89 (0.00, 3.77) | 0.142 | 0.75 (0.00, 1.94) | 0.60 (0.00, 2.04) | 0.87 (0.00, 1.89) | 0.860 |
| Scyllo-inositol / (Cr+PCr) [×10^-3^] | 11.00 (0.00, 22.00) | 12.00 (2.15, 20.50) | 5.15 (0.00, 22.00) | 0.198 | 5.60 (0.00, 12.00) | 4.10 (0.00, 12.00) | 6.60 (0.00, 13.50) | 0.640 |
| Taurine conc. [×10^-6^] | 8.15 (0.01, 17.30) | 5.58 (0.00, 15.75) | 10.20 (2.00, 19.05) | 0.199 | 1.40 (0.00, 7.15) | 0.00 (0.00, 4.29) | 3.06 (0.00, 8.84) | **0.018** |
| Taurine / (Cr+PCr) | 0.05 (0.00, 0.10) | 0.03 (0.00, 0.08) | 0.06 (0.01, 0.11) | 0.093 | 10.00 (0.00, 55.00) | 0.00 (0.00, 27.00) | 22.50 (0.00, 68.00) | **0.009** |
| (Cr+PCr) conc. [×10^-6^] | 176.00 (165.00, 188.00) | 182.00 (172.50, 195.00) | 168.00 (161.25, 179.00) | **<0.001** | 144.00  (134.00, 154.00) | 150.00  (140.50, 159.50) | 137.50  (129.50, 150.00) | **0.002** |
| (Cr+PCr) / (Cr+PCr) | 1.00 (1.00, 1.00) | 1.00 (1.00, 1.00) | 1.00 (1.00, 1.00) | 1.000 | 1.00 (1.00, 1.00) | 1.00 (1.00, 1.00) | 1.00 (1.00, 1.00) | 1.000 |
| (Glu+Gln) conc. [×10^-6^] | 394.00 (362.00, 428.00) | 426.00 (391.50, 443.50) | 368.50 (339.75, 407.50) | **<0.001** | 315.00  (285.00, 336.00) | 328.00  (296.00, 343.50) | 300.50  (283.25, 324.50) | 0.057 |
| (Glu+Gln) / (Cr+PCr) | 2.27 (2.07, 2.39) | 2.35 (2.19, 2.42) | 2.18 (2.02, 2.34) | **0.014** | 2.14 (2.03, 2.29) | 2.14 (2.02, 2.26) | 2.16 (2.09, 2.33) | 0.126 |
| Lip13a conc. [×10^-6^] | 86.70 (38.70, 117.00) | 93.70 (54.25, 112.00) | 75.25 (34.85, 128.50) | 0.574 | 21.10 (5.12, 56.70) | 16.10 (5.34, 68.25) | 21.10 (4.93, 48.15) | 0.563 |
| Lip13a / (Cr+PCr) | 0.47 (0.22, 0.70) | 0.47 (0.29, 0.60) | 0.44 (0.20, 0.77) | 0.917 | 0.14 (0.04, 0.38) | 0.09 (0.04, 0.46) | 0.14 (0.03, 0.35) | 0.749 |
| Lip20 conc. [×10^-6^] | 9.65 (4.87, 15.30) | 11.00 (5.99, 14.70) | 9.27 (3.15, 15.75) | 0.432 | 1.88 (0.00, 6.67) | 3.54 (0.83, 7.98) | 1.44 (0.00, 4.62) | **0.025** |
| Lip20 / (Cr+PCr) | 0.05 (0.03, 0.08) | 0.06 (0.03, 0.08) | 0.05 (0.02, 0.09) | 0.761 | 13.00 (0.00, 43.00) | 24.00 (5.30, 56.50) | 9.35 (0.00, 32.75) | **0.036** |
| (Lip13a+Lip13b) conc. [×10^-6^] | 86.70 (39.50, 117.00) | 93.70 (54.25, 112.00) | 75.25 (36.92, 128.50) | 0.587 | 22.70 (8.13, 56.70) | 37.50 (9.09, 68.25) | 21.90 (6.62, 48.50) | 0.202 |
| (Lip13a+Lip13b) / (Cr+PCr) | 0.47 (0.23, 0.70) | 0.47 (0.29, 0.60) | 0.44 (0.21, 0.77) | 0.975 | 0.16 (0.05, 0.42) | 0.23 (0.06, 0.46) | 0.15 (0.04, 0.35) | 0.309 |
| Missing values | 3 | 2 | 1 |  | 3 | 2 | 1 |  |
| *^1^* *Mdn* (*Q1, Q3*) | | | | |  |  |  |  |
| *^2^* Wilcoxon rank sum test | | | | |  |  |  |  |

Supplementary Table S4. Krzyściak Wirginia, Title: Statistical Predictive Model of Schizophrenia. Results of metabolites assessment in anterior cingulate cortex (ACC) and posterior cingulate cortex (PCC) at echo time (TE) 30 ms. Distribution of results by groups and for the entire sample.

| *Characteristic* | *Overall,*  *N = 96^1^* | *ACC*  *Group* | | *p* | *Overall,*  *N = 96^1^* | *PCC*  *Group* | | *p* |
| --- | --- | --- | --- | --- | --- | --- | --- | --- |
|  |  | *control, n = 45^1^* | *test, n = 51^1^* |  |  | *control, n = 45^1^* | *test, n = 51^1^* |  |
| L-alanine conc. | 7.69 (0.00, 38.04) | 12.90 (0.00, 50.35) | 0.22 (0.00, 29.85) | 0.168 | 9.43 (0.00, 38.26) | 22.79 (0.00, 53.39) | 0.00 (0.00, 33.70) | **0.046** |
| L-alanine / (Cr+PCr) | 0.01 (0.00, 0.04) | 0.01 (0.00, 0.05) | 0.00 (0.00, 0.03) | 0.239 | 12.00 (0.00, 52.00) | 28.00 (0.00, 64.00) | 0.00 (0.00, 42.50) | 0.060 |
| Creatine conc. | 462.58 (407.06, 527.46) | 464.79 (409.16, 545.86) | 459.77 (396.25, 520.97) | 0.697 | 327.34 (243.14, 396.04) | 295.14 (212.61, 387.11) | 355.40 (276.74, 406.85) | 0.169 |
| Creatine / (Cr+PCr) | 0.45 (0.40, 0.52) | 0.44 (0.39, 0.50) | 0.46 (0.41, 0.55) | 0.339 | 0.42 (0.31, 0.50) | 0.39 (0.29, 0.47) | 0.45 (0.38, 0.53) | **0.027** |
| Phosphocreatine conc. | 577.34 (472.00, 626.03) | 583.00 (524.79, 634.57) | 540.98 (430.93, 618.37) | **0.036** | 462.20 (385.90, 560.01) | 492.69 (425.57, 583.56) | 427.97 (369.32, 504.77) | **0.004** |
| Phosphocreatine / (Cr+PCr) | 0.55 (0.48, 0.59) | 0.56 (0.50, 0.61) | 0.54 (0.45, 0.59) | 0.282 | 0.58 (0.51, 0.69) | 0.61 (0.53, 0.71) | 0.55 (0.47, 0.62) | **0.027** |
| Glutamine (Gln) conc. | 101.01 (24.41, 181.54) | 104.28 (5.37, 174.96) | 96.91 (35.48, 216.29) | 0.699 | 29.97 (0.00, 125.62) | 22.49 (0.00, 163.95) | 31.66 (0.00, 97.76) | 0.765 |
| Glutamine / (Cr+PCr) | 0.10 (0.03, 0.20) | 0.10 (0.00, 0.18) | 0.10 (0.04, 0.25) | 0.322 | 0.04 (0.00, 0.17) | 0.02 (0.00, 0.21) | 0.04 (0.00, 0.15) | 0.830 |
| Glutamate (Glu) conc. | 1,020.00 (845.75, 1,160.00) | 1,130.00 (910.22, 1,250.00) | 940.17 (792.23, 1,087.50) | **<0.001** | 500.27 (391.50, 629.40) | 526.36 (392.13, 615.37) | 487.78 (376.00, 631.86) | 0.603 |
| Glutamate / (Cr+PCr) | 1.01 (0.85, 1.10) | 1.07 (0.91, 1.18) | 0.97 (0.81, 1.05) | **0.004** | 0.63 (0.49, 0.81) | 0.63 (0.49, 0.81) | 0.64 (0.48, 0.80) | 0.978 |
| Glycerophosphocholine (GPC) conc. | 152.37 (0.00, 334.85) | 105.00 (0.00, 283.58) | 180.18 (103.41, 335.39) | 0.201 | 167.45 (122.62, 195.33) | 174.04 (154.49, 195.51) | 160.47 (122.33, 192.49) | 0.622 |
| Glycerophosphocholine / (Cr+PCr) | 0.14 (0.00, 0.32) | 0.10 (0.00, 0.28) | 0.18 (0.10, 0.32) | 0.084 | 0.22 (0.15, 0.25) | 0.23 (0.14, 0.24) | 0.22 (0.16, 0.26) | 0.608 |
| Phosphocholine (PCh) conc. | 207.13 (23.35, 299.00) | 235.73 (70.80, 350.35) | 154.74 (26.40, 265.66) | 0.093 | 0.00 (0.00, 82.09) | 0.00 (0.00, 82.94) | 0.14 (0.00, 81.29) | 0.753 |
| Phosphocholine / (Cr+PCr) | 0.19 (0.02, 0.31) | 0.23 (0.07, 0.35) | 0.15 (0.03, 0.26) | 0.197 | 0.00 (0.00, 0.11) | 0.00 (0.00, 0.09) | 0.00 (0.00, 0.12) | 0.706 |
| Glutathione conc. | 35.43 (9.79, 71.53) | 33.41 (5.03, 63.29) | 46.34 (16.43, 72.24) | 0.320 | 41.73 (10.26, 72.54) | 30.85 (2.75, 79.71) | 46.52 (20.33, 70.92) | 0.731 |
| Glutathione / (Cr+PCr) | 0.04 (0.01, 0.07) | 0.03 (0.00, 0.06) | 0.05 (0.02, 0.07) | 0.286 | 56.00 (13.00, 94.00) | 41.00 (3.55, 100.50) | 62.50 (28.00, 89.00) | 0.612 |
| N-Acetylaspartate conc. | 1,510.00  (1,390.00, 1,640.00) | 1,580.00  (1,505.00, 1,700.00) | 1,440.00  (1,322.50, 1,557.50) | **<0.001** | 1,340.00  (1,250.00, 1,420.00) | 1,350.00  (1,290.00, 1,460.00) | 1,325.00  (1,235.00, 1,415.00) | 0.115 |
| N-Acetylaspartate / (Cr+PCr) | 1.49 (1.38, 1.59) | 1.53 (1.45, 1.63) | 1.44 (1.36, 1.56) | **0.011** | 1.73 (1.60, 1.85) | 1.73 (1.59, 1.82) | 1.73 (1.61, 1.87) | 0.515 |
| N-Acetylaspartylglutamate conc. | 65.43 (24.32, 107.59) | 74.00 (28.49, 113.68) | 61.42 (21.36, 91.28) | 0.217 | 59.49 (14.79, 103.26) | 62.75 (20.16, 105.37) | 51.12 (9.99, 100.44) | 0.548 |
| N-Acetylaspartylglutamate / (Cr+PCr) | 0.07 (0.02, 0.11) | 0.07 (0.02, 0.11) | 0.06 (0.02, 0.09) | 0.400 | 0.07 (0.02, 0.14) | 0.07 (0.02, 0.13) | 0.07 (0.01, 0.13) | 0.609 |
| Scylloinositol conc. | 0.00 (0.00, 10.95) | 0.00 (0.00, 14.85) | 0.00 (0.00, 6.23) | 0.317 | 4.92 (0.00, 18.96) | 13.62 (0.85, 21.16) | 0.18 (0.00, 13.93) | **0.005** |
| Scylloinositol / (Cr+PCr) | 0.00 (0.00, 0.01) | 0.00 (0.00, 0.01) | 0.00 (0.00, 0.01) | 0.337 | 0.01 (0.00, 0.02) | 0.02 (0.00, 0.03) | 0.00 (0.00, 0.02) | **0.009** |
| Taurine conc. | 63.72 (13.30, 139.57) | 96.55 (13.32, 150.94) | 50.98 (13.63, 123.00) | 0.190 | 61.31 (0.75, 102.15) | 68.01 (13.41, 113.62) | 48.92 (0.00, 82.89) | 0.105 |
| Taurine / (Cr+PCr) | 0.07 (0.02, 0.13) | 0.09 (0.02, 0.13) | 0.05 (0.01, 0.12) | 0.266 | 0.08 (0.00, 0.13) | 0.09 (0.02, 0.14) | 0.06 (0.00, 0.11) | 0.146 |
| CrCH_2_ conc. | 0.00 (0.00, 21.13) | 0.00 (0.00, 30.69) | 0.00 (0.00, 14.72) | 0.728 | 21.95 (0.00, 64.75) | 12.62 (0.00, 63.07) | 27.90 (0.00, 65.30) | 0.585 |
| CrCH_2_ / (Cr+PCr) | 0.00 (0.00, 0.02) | 0.00 (0.00, 0.03) | 0.00 (0.00, 0.02) | 0.763 | 0.03 (0.00, 0.09) | 0.02 (0.00, 0.08) | 0.04 (0.00, 0.09) | 0.500 |
| (Glycerophosphocholine (GPC) + Phosphocholine (PCh)) conc. | 372.55 (339.39, 402.38) | 376.80 (347.55, 404.24) | 369.74 (337.84, 396.78) | 0.373 | 196.40 (180.32, 215.69) | 191.71 (178.79, 211.15) | 202.96 (183.03, 225.75) | 0.243 |
| (Glycerophosphocholine (GPC) + Phosphocholine (PCh)) / (Cr+PCr) | 0.37 (0.34, 0.39) | 0.36 (0.34, 0.38) | 0.38 (0.34, 0.40) | 0.087 | 0.25 (0.23, 0.28) | 0.24 (0.23, 0.26) | 0.26 (0.24, 0.29) | **0.002** |
| (N-Acetyaspartate + N-Acetylspartylglutamate) conc. | 1,600.00 (1,470.00, 1,730.00) | 1,680.00 (1,585.00, 1,775.00) | 1,510.00 (1,372.50, 1,620.00) | **<0.001** | 1,400.00  (1,290.00, 1,500.00) | 1,430.00  (1,355.00, 1,495.00) | 1,360.00  (1,260.00, 1,495.00) | 0.078 |
| (N-Acetyaspartate + N-Acetylspartylglutamate) / (Cr+PCr) | 1.58 (1.48, 1.66) | 1.61 (1.56, 1.71) | 1.50 (1.43, 1.63) | **0.006** | 1.84 (1.68, 1.94) | 1.85 (1.63, 1.94) | 1.83 (1.70, 1.93) | 0.752 |
| (Cr+PCr) conc. | 1,020.00  (951.47, 1,090.00) | 1,050.00  (974.44, 1,130.00) | 991.63  (924.55, 1,065.00) | **0.004** | 787.30 (716.72, 854.82) | 798.19 (722.11, 888.32) | 776.09 (707.24, 825.04) | 0.115 |
| (Cr+PCr) / (Cr+PCr) | 1.00 (1.00, 1.00) | 1.00 (1.00, 1.00) | 1.00 (1.00, 1.00) | 0.365 | 1.00 (1.00, 1.00) | 1.00 (1.00, 1.00) | 1.00 (1.00, 1.00) |  |
| (Glu+Gln) conc. | 1,150.00  (1,000.00, 1,310.00) | 1,240.00  (1,135.00, 1,360.00) | 1,095.00  (910.91, 1,237.50) | **0.001** | 564.95 (448.49, 723.41) | 612.82 (507.72, 756.22) | 522.82 (437.33, 679.95) | 0.132 |
| (Glu+Gln) / (Cr+PCr) | 1.13 (1.04, 1.25) | 1.20 (1.08, 1.31) | 1.11 (0.99, 1.19) | **0.009** | 0.73 (0.61, 0.93) | 0.78 (0.61, 0.97) | 0.70 (0.61, 0.88) | 0.430 |
| Lip13a conc. | 0.00 (0.00, 0.00) | 0.00 (0.00, 0.00) | 0.00 (0.00, 0.00) | 1.00 | 0.00 (0.00, 0.01) | 0.00 (0.00, 0.00) | 0.00 (0.00, 0.60) | 0.222 |
| Lip13a / (Cr+PCr) | 0.00 (0.00, 0.00) | 0.00 (0.00, 0.00) | 0.00 (0.00, 0.00) | 0.987 | 0.00 (0.00, 0.00) | 0.00 (0.00, 0.00) | 0.00 (0.00, 0.00) | 0.215 |
| Lip20 conc. | 5.06 (0.00, 21.33) | 2.89 (0.00, 18.65) | 6.00 (0.00, 22.59) | 0.425 | 0.53 (0.00, 11.37) | 0.53 (0.00, 6.25) | 0.72 (0.00, 11.77) | 0.675 |
| Lip20 / (Cr+PCr) | 0.01 (0.00, 0.02) | 0.00 (0.00, 0.02) | 0.01 (0.00, 0.02) | 0.357 | 0.66 (0.00, 13.00) | 0.66 (0.00, 7.90) | 0.96 (0.00, 17.50) | 0.582 |
| (Lip13a+Lip13b) conc. | 22.70 (0.00, 126.18) | 15.96 (0.00, 115.12) | 28.05 (0.00, 134.03) | 0.903 | 1.38 (0.00, 71.83) | 1.38 (0.00, 40.40) | 1.99 (0.00, 72.99) | 0.934 |
| (Lip13a+Lip13b) / (Cr+PCr) | 0.02 (0.00, 0.12) | 0.02 (0.00, 0.10) | 0.03 (0.00, 0.14) | 0.839 | 2.00 (0.00, 81.00) | 2.00 (0.00, 47.00) | 2.55 (0.00, 104.00) | 0.875 |
| Missing values | 3 | 2 | 1 |  | 3 | 2 | 1 |  |
| *^1^* *Mdn* (*Q1, Q3*) | | | | |  |  |  |  |
| *^2^* Wilcoxon rank sum test | | | | |  |  |  |  |

Supplementary Table S5. Krzyściak Wirginia, Title: Statistical Predictive Model of Schizophrenia. Results of metabolites assessment in anterior cingulate cortex (ACC) and posterior cingulate cortex (PCC) at echo time (TE) 144 ms. Distribution of results by groups and for the entire sample.


| *Predictor* | *Marginal effect* | | | *SE* | *z* | *p* |
| --- | --- | --- | --- | --- | --- | --- |
|  | *AME* | *CI95%* | |  |  |  |
|  |  | *ll* | *ul* |  |  |  |
| age | -0.09 | -0.19 | 0.01 | 0.05 | -1.82 | 0.068 |
| sex [male] | -1.10 | -3.51 | 1.31 | 1.23 | -0.89 | 0.371 |
| BDI II score | 0.11 | 0.03 | 0.18 | 0.04 | 2.68 | 0.007 |
| Glucose | 3.22 | -0.06 | 0.25 | 1.31 | 2.46 | 0.014 |
| Insulin | 0.10 | -0.06 | 0.25 | 0.08 | 1.21 | 0.227 |
| (Cr+PCr) conc^1^ | -0.10 | -0.18 | -0.03 | 0.04 | -2.66 | 0.008 |
| Taurine / (Cr+PCr)*^1^* | 0.03 | 0.01 | 0.05 | 0.01 | 2.48 | 0.013 |
| RBC | 2.05 | -0.69 | 4.79 | 1.40 | 1.46 | 0.143 |
| Re-lymph | 47.37 | 14.91 | 79.83 | 16.56 | 2.85 | 0.004 |
| WBC | 0.33 | -0.18 | 0.85 | 0.26 | 1.27 | 0.202 |

*Note:* ^1^—rear rim turn TE 30 ms. BDI II, Beck Depression Inventory; Cr PCr conc, sum of concentrations of creatine and phosphocreatine; RBC, red blood cells; Re-Lymph, reactive lymphocytes; WBC, white blood cells.

Supplementary Table S6. Krzyściak Wirginia, Title: Statistical Predictive Model of Schizophrenia*.* Estimation of the average marginal effects of the fitted model. From presented table, the following conclusions were drawn:

The estimation of average marginal effects (AMEs) for the fitted logistic regression model provided a nuanced understanding of how each predictor influenced the probability of schizophrenia occurrence, with each AME representing the average change in predicted probability associated with a one-unit change in the predictor variable, holding other variables constant.

The negative AME for age (AME = -0.09, *p* = 0.068) suggested a slight decrease in the probability of schizophrenia occurrence with each additional year. Although this effect approaches but did not reach conventional levels of statistical significance, it was consistent with the known peak age of onset for schizophrenia during late adolescence and early adulthood.

The AME for sex indicated that being male was associated with a substantial decrease in the probability of schizophrenia occurrence (AME = -1.10, *p* = 0.371), though this finding was not statistically significant. This contrasted with some epidemiological data which suggest a higher prevalence and an earlier age of onset in males, indicating that the effect of sex on schizophrenia risk may be complex and potentially influenced by factors not captured in this model.

The positive AME of the BDI II score (AME = 0.11, *p* = 0.007) was statistically significant and suggested that higher levels of depressive symptoms were associated with an increased probability of schizophrenia. This highlighted the importance of mood symptomatology as a component of the schizophrenia spectrum and may reflect the negative symptom domain of the disorder or a comorbid depressive disorder.

The AME for glucose was positive (AME = 3.22, *p* = 0.014), indicating that higher glucose levels were significantly associated with an increased probability of schizophrenia. This supported the hypothesis of metabolic dysregulation in schizophrenia and aligns with the literature on glucose abnormalities in the disorder, potentially reflecting insulin resistance or altered glucose metabolism.

Insulin levels have a non-significant positive AME (AME = 0.10, *p* = 0.227), suggesting a possible association with increased schizophrenia probability, though this effect was not statistically robust. This may underscore the relevance of insulin signaling pathways in schizophrenia, particularly given the metabolic side effects of antipsychotic medications.

The AME for the sum of creatine and phosphocreatine concentrations was negative and statistically significant (AME = -0.10, *p* = 0.008), suggesting that higher concentrations were associated with a reduced probability of schizophrenia. This finding implicated energy metabolism alterations in schizophrenia pathophysiology and may reflect mitochondrial dysfunction or altered energy reserve capacity in the disorder.

A positive significant AME (AME = 0.03, *p* = 0.013) for the taurine to (Cr+PCr) ratio suggests that an increased ratio was associated with a higher probability of schizophrenia. This could relate to the role of taurine as a neuromodulator and its involvement in neurotransmission and osmoregulation.

The AME for RBC count was positive but not statistically significant (AME = 2.05, *p* = 0.143), indicating a non-robust association with the probability of schizophrenia. The clinical relevance of this finding is uncertain, but it may warrant further investigation.

The AME for reactive lymphocytes showed a substantial positive association with schizophrenia (AME = 47.37, *p* = 0.004), suggesting a strong link between immune response and the disorder. This may reflect an underlying inflammatory process in schizophrenia pathogenesis.

Lastly, the AME for WBC count was positive (AME = 0.33, *p* = 0.202) but not significant, which did not provide strong evidence for inflammation as a factor in schizophrenia within this sample, although it did not rule out its potential involvement.

| *Predictor* | *Optimal cutpoint* | *Accuracy* | *Sensitivity* | *Specificity* | *AUC* |
| --- | --- | --- | --- | --- | --- |
| WBC | 7.34 | 0.65 | 0.47 | 0.84 | 0.67 |
| Re-lymph | 0.07 | 0.69 | 0.55 | 0.84 | 0.73 |
| RBC | 4.92 | 0.68 | 0.57 | 0.80 | 0.68 |
| Glucose | 4.66 | 0.67 | 0.71 | 0.62 | 0.71 |
| Insulin | 10.6 | 0.71 | 0.67 | 0.76 | 0.73 |
| BDI II score | 19.0 | 0.63 | 0.45 | 0.86 | 0.64 |
| Taurine/(Cr+PCr)*^1^* | 37.0 | 0.62 | 0.48 | 0.79 | 0.65 |
| Cr+PCr conc*^1^* | 147.0 | 0.69 | 0.65 | 0.72 | 0.69 |

*Note:* ^1^—rear rim turn TE 30 ms. WBC, white blood cells; Re-Lymph, reactive lymphocytes; RBC, red blood cells; BDI II, Beck Depression Inventory; ratio of Taurine to sum of concentrations of creatine and phosphocreatine, creatine and phosphocreatine (Cr+PCr) concentration.

Supplementary Table S7. Krzyściak Wirginia, Title: Statistical Predictive Model of Schizophrenia. Estimation of the cutoff points for each exposure in the final regression model.

| *term* | *VIF* | *CI 95%* |
| --- | --- | --- |
| Sex | 2.66 | 0.28 – 0.49 |
| Age | 1.58 | 0.47 – 0.77 |
| WBC | 1.44 | 0.52 – 0.83 |
| Re-Lymph | 1.77 | 0.42 – 0.70 |
| RBC | 2.44 | 0.30 – 0.53 |
| Glucose | 1.60 | 0.47 – 0.76 |
| Insulin | 1.41 | 0.53 – 0.84 |
| BDI II | 1.85 | 0.40 – 0.67 |
| (Cr+PCr) conc | 1.60 | 0.47 – 0.76 |
| Taurine / (Cr+PCr) | 2.30 | 0.32 – 0.55 |

*Note:* *VIF* – Variance Inflation Factor;  *95% CI* – VIF Confidence Interval 95%.

Supplementary Table S8. Krzyściak Wirginia, Title: Statistical Predictive Model of Schizophrenia. Results of VIF analysis for regression model.

Results and interpretation of the cross-validation procedure.

The fitted regression model was evaluated using a 10-fold cross-validation technique, a resampling method that provides a robust estimate of the model's predictive accuracy by partitioning the original sample into ten equally sized subsamples, using nine for training and one for validation, iteratively. The reported accuracy of the model was 81.22%, indicating a high level of overall correctness in the model's predictions when generalizing to unseen data. This suggested that the predictors included in the model captured a substantial amount of the variance related to the outcome of interest.

The Kappa statistic, or Cohen's Kappa, which adjusted accuracy by accounting for the possibility of correct predictions by chance, was 0.62. A Kappa value above 0.60 is generally considered to indicate 'substantial' agreement according to Landis and Koch's benchmarks. This implied that the model's predictive performance was significantly better than random chance, further underscoring the potential relevance of the predictors used.

From a clinical perspective, the model's performance suggested that the 8 predictors (adjusted with age and sex) can be useful in differentiating between patients with schizophrenia and control.

Results of impact of olanzapine intake on designated clinical parameters

In this study, we employed an ordinary least squares (OLS) linear regression model, as outlined in equations (1) and (2), to estimate the adjusted impact of olanzapine intake on designated clinical parameters. The rationale for utilizing regression analysis was to concurrently scrutinize the influence of multiple variables on the outcome. This analytical approach enabled a comprehensive exploration of how these variables, both individually and collectively, affect the outcome of interest.

| *Characteristic* | *N* | *Taking olanzapine* | | *p* |
| --- | --- | --- | --- | --- |
|  |  | *no,*  *n = 25^1^* | *yes,*  *n = 26^1^* |  |
| Gender: | 51 |  |  | 0.332*^5^* |
| female |  | 12.0 (48.0%)*^2^* | 9.0 (34.6%)*^2^* |  |
| male |  | 13.0 (52.0%)*^2^* | 17.0 (65.4%)*^2^* |  |
| Age, years | 51 | 32.0 (25.0, 36.0) | 23.5 (17.2, 34.0) | *0.066* |
| WBC [×10^3^/µL] | 51 | 7.3 (6.3, 8.3) | 6.7 (5.6, 8.6) | 0.407 |
| Re-Lymph [×10^6^/µL] | 51 | 80.0 (60.0, 140.0) | 60.0 (32.5, 70.0) | **0.008** |
| RBC [×10^6^/µL] | 51 | 4.93 (0.45) *^3^* | 5.00 (0.47) *^3^* | 0.596*^6^* |
| Taurine/Cr+PCr ×10^3^  (rear rim turn TE 30ms) | 50 | 23.6 (0.0, 70.5) | 22.5 (3.4, 55.5) | 0.600 |
| *Missing values* |  | *1* | *0* |  |
| Cr+PCr conc. ×10^6^  (rear rim turn TE 30ms) | 50 | 137.79 (17.84) *^3^* | 141.73 (14.90) *^3^* | 0.403*^6^* |
| *Missing values* |  | *1* | *0* |  |
| Insulin [µU/mL] | 51 | 12.0 (8.4, 23.9) | 11.9 (9.8, 15.2) | 0.685 |
| Glucose [mmol/l] | 51 | 5.0 (4.7, 5.3) | 4.7 (4.5, 5.2) | 0.474 |
| BDI II total score | 46 | 11.0 (5.0, 24.0) | 22.0 (7.0, 31.0) | 0.173 |
| *Missing values* |  | *2* | *3* |  |
| Effective dose of all drugs per olanzapine [mg] | 51 | 13.9 (10.0, 23.9) | 22.0 (15.0, 29.7) | **0.035** |
| *^1^ Mdn* (*Q1*, *Q3*)  *^2^ n* (%);  *^3^ M* (*SD*); | | | | |
| *^4^* Wilcoxon rank sum test;  *^5^* Pearson's Chi-squared test;  *^6^* t -Welch test | | | | |

Supplementary Table S9. Krzyściak Wirginia, Title: Statistical Predictive Model of Schizophrenia. Distributions of the studied parameters with the results of the statistical tests examining the differences between the groups depending on the olanzapine intake.

*The significance and magnitude of the effect of taking olanzapine on eight continuous clinical parameters among patients with schizophrenia.*

In our model, we considered olanzapine as an independent variable and adjusted for potential confounders such as the Effective Dose of all Drugs per Olanzapine (EDDO), gender, and age. In order to capture potential synergistic or antagonistic effects between the predictors, interaction terms were systematically integrated into the model. This comprehensive modelling approach allowed us to discern the subtle and complex relationships between these variables and their collective impact on the clinical parameters under investigation.

*Yi = β₀ + β₁·age_i_ + β₂·EDDO_i_ + β₃·gender_i_ + β_4_·olanzapine_i_ + β_5_·EDDO_i_× gender_i_ +*

*β_6_·EDDO_i_ × olanzapine_i_ + β_7_·gender_i_ × olanzapine_i_ + β_8_ EDDO_i_ × gender_i_ × olanzapine_i_ + ε_i_ (1), ε ~ N(0,σ^2^), (2)*

where *Y_i_* – represented the dependent variable, olanzapine was the modifier, the EDDO (Effective Dose of all Drugs per Olanzapine), gender and age were the confounders; β₀ –represented the intercept term, which is the estimated mean *Y_i_* when all the independent confounding variables were zero (for continuous) or at reference category; β₁ - β₈, were the regression coefficients associated with each independent variable. These coefficients represented the expected change in the *Yi* for a one-unit change (for continuous) or for change in category (for dichotomous) in the corresponding independent variable, holding other variables constant; ε represented the error term or the residuals, which captured the unexplained variation *σ^2^* in the *Y_i_* not accounted for by the independent variables.

The determination of both the significance and the magnitude of the effects associated with olanzapine intake was carried out by employing the Ordinary Least Squares (OLS) estimator. This analysis scrutinized the regression coefficients (β₁ through β₈), thereby providing a quantitative appraisal of the influence of olanzapine on the variables under consideration. Hypothesis testing was performed to assess the statistical significance of the coefficients, and confidence intervals (CI) were calculated to estimate the range of the effects. The 95% CIs and *p*-values were computed using a Wald *t-*distribution approximation. The assumption of a normal distribution for the error term (ε) was also verified.

The study sample consisted of N=51 participants, with 49% (n1 = 25) not taking olanzapine and 51% (n2 = 26) taking olanzapine as part of their treatment for schizophrenia. The study included eight parameters that have been previously identified as predictors of schizophrenia occurrence in the sample healthy individuals. These parameters encompass various blood count metrics (WBC, Re-Lymph, RBC), brain parameters related to the rear rim turn TE 30ms (taurine/Cr+PCr, Cr+PCr conc.), insulin and glucose levels, as well as the total score from the BDI II questionnaire. Additionally, the effective dose of all medications, including olanzapine, was documented, along with patients' sociodemographic data such as gender and age.

Supplementary Table S9 presented a comparison of the distribution of the studied parameters between the two groups: patients taking olanzapine and those not taking olanzapine. Statistical significance testing was performed to assess the differences between the two groups, with corresponding p-values reported.

The comparative analysis of patients with schizophrenia, delineated in Supplementary Table S9, verified certain key associations with the administration of olanzapine. The demographic parameters encompassed gender distribution and age. These parameters did not exhibit a statistically significant difference (p > 0.05) between the two cohorts, indicating that the decision to administer olanzapine in this patient population was ostensibly not influenced by demographic factors like gender and age. Investigation into white blood cell (WBC) count unveiled no significant disparity between the two cohorts. This finding suggested that olanzapine administration may not substantially modulate WBC count in this patient population. In contrast, relative lymphocyte count demonstrated a significant difference between the two cohorts. The median Re-lymph count in the olanzapine cohort was markedly lower compared to the patients not receiving olanzapine. This observation inferred a potential association between olanzapine administration and the immunological profile of patients, specifically influencing lymphocyte levels. Several physiological parameters, including red blood cell count (RBC), taurine/Cr+PCr, Cr+PCr conc., insulin levels, and glucose levels, did not exhibit significant disparities between the two cohorts. These findings suggested that the administration of olanzapine may not significantly impinge upon these physiological parameters. Interestingly, a significant deviation was observed in the effective dose of all drugs per olanzapine between the two cohorts. The patients receiving olanzapine demonstrated a higher median effective dose compared to their counterparts not receiving olanzapine. This observation implied a more intensive therapeutic regimen in patients receiving olanzapine, potentially reflective of a more severe or complex clinical condition.

In summary, the treatment regimen involving olanzapine may be associated with lower reactive lymphocyte counts, suggesting potential immunological implications that warrant further exploration. However, this regimen did not appear to significantly influence other examined physiological parameters. The higher EDDO underscored a more comprehensive therapeutic approach in patients receiving olanzapine, potentially indicative of a more intricate clinical scenario.

*Estimation of the effects of taking olanzapine and the effective dose of all drugs taken per olanzapine on selected parameters*

WBC [×10^3^/µL]

The application OLS estimator was employed to analyze the effects of various predictors according equations (1), (2) on the WBC count (expressed in ×10^3^/µL). The estimates, along with their 95% CIs and associated p-values, were reported in Supplementary Table S10. The model explained only 12.6% of the variance in the outcome variable.

The multivariate regression model indicated that none of the examined predictors (age, EDDO, gender, and olanzapine intake) nor their interactions were associated with a significant influence on the WBC count in the studied patient population. This suggested that the WBC count of patients with schizophrenia may not be significantly affected by these factors, including the intake of olanzapine.

Detailed interpretation:

The intercept of the model, representing the predicted WBC concentration for a reference individual (i.e., an individual aged zero with EDDO = 0 mg, not taking olanzapine, female), was found to be *B*_0_ = 6.64 (*95% CI*: 3.77 – 9.52, *p* <0.001), suggesting a significant baseline WBC level.

The age of the patient did not present a significant influence on the WBC, with an estimate of *B_1_* = 0.04 (*95% CI*: -0.05 – 0.12, *p* = 0.380). Similarly, the EDDO also did not significantly affect the WBC count (*B_2_*= 0.02, *95% CI*: -0.05 – 0.09, *p* = 0.533). Gender, indicated by being male, did not have a significant effect on the WBC level (*B_3_* = -0.60, *95% CI*: -3.97 – 2.77, *p* = 0.721). The intake of olanzapine did not significantly affect the WBC level either (*B_4_* = -0.44, *95% CI*: -4.27 – 3.39, *p* = 0.819).

The interaction terms involving EDDO and gender, EDDO and olanzapine, gender and olanzapine, and all three predictors (EDDO, gender, and olanzapine) also did not demonstrate a significant influence on the WBC count (*p* > 0.05).

| *Predictors* | **WBC [×10^3^/µL]** | | |
| --- | --- | --- | --- |
|  | *Estimates* | *CI 95%* | *p* |
| (Intercept) | 6.64 | 3.77 – 9.52 | **<0.001** |
| age | 0.04 | -0.05 – 0.12 | 0.380 |
| EDDO [mg] | 0.02 | -0.05 – 0.09 | 0.533 |
| gender [male] | -0.60 | -3.97 – 2.77 | 0.721 |
| olanzapine [yes] | -0.44 | -4.27 – 3.39 | 0.819 |
| EDDO × gender [male] | -0.06 | -0.27 – 0.16 | 0.604 |
| EDDO × olanzapine [yes] | -0.05 | -0.21 – 0.11 | 0.519 |
| gender [male] × olanzapine [yes] | 0.67 | -4.94 – 6.27 | 0.812 |
| EDDO × gender[male] × olanzapine [yes] | 0.11 | -0.18 – 0.40 | 0.464 |

Supplementary Table S10. Krzyściak Wirginia, Title: Statistical Predictive Model of Schizophrenia. Results of fitting regression model with WBC as outcome, *n_obs_* = 51

### Re-Lymph [×10^6^/µL]

The estimates, along with their 95% CI and associated *p*-values, were reported in Supplementary Table S11. The model's goodness of fit, *R^2^* = 0.583, indicating that approximately 58.3% of the variance in relative lymphocyte count (Re-Lymph) can be accounted for by the predictors used in the model. The adjusted R^2^ value, which takes into consideration the number of predictors used, was 0.504.

Our study has highlighted that re-lymph in patients was influenced by multiple factors and their interactions, including age, gender, EDDO, and olanzapine intake. Age and being male showed a borderline significant impact, while EDDO demonstrated a significant positive effect on the Re-Lymph count. Although olanzapine intake did not significantly affect the Re-Lymph count overall, its interaction with EDDO and gender was significant. Specifically, among females with an EDDO of 19.6 mg, olanzapine use was associated with a significantly lower Re-Lymph count, while this was not the case in males.

Detailed interpretation:

The age of the patient presented a borderline significance in influencing the re-lymph count, with an estimate of B_1_ = 1.24×10^3^/µL (*95% CI*: -0.23 – 2.71, *p* = 0.096). The EDDO had a significant positive effect on the Re-lymph count, B_2_ = 2.78×10^3^/µL (*95% CI*: 1.59 – 3.97, *p* < 0.001). The gender effect, as indicated by being male, showed a borderline significance, with a positive estimate of B_3_ = 55.08 ×10^3^/µL (*95% CI*: -3.06 – 113.21, *p* = 0.063). Olanzapine intake did not significantly affect the Re-Lymph count, B_4_ = 14.28×10^3^/µL (*95% CI*: -51.74 – 80.30, *p* = 0.665).

The interaction terms between EDDO and gender, demonstrated a statistically significant influence on the re-lymph count. Specifically, at an EDDO level of 19.6 mg, the re-lymph concentration in males was significantly lower than that in females by an estimated B_5_ = -6.11 × 10^3^/µL (*95% CI*: -9.84 – -2.38, *p* = 0.002).

The interaction terms between EDDO and olanzapine, also demonstrated a statistically significant influence on the Re-Lymph count. More specifically, for patients at an EDDO level of 19.6 mg, the intake of olanzapine was associated with a significant reduction in Re-Lymph concentration. Patients who were taking olanzapine had, on average, a re-lymph count that was lower by an estimated B_6_ = -3.39 × 10^3^/µL (*95% CI*: -6.15 – -0.63, *p* = 0.017) compared to those not taking olanzapine. This finding was statistically significant, as indicated by the p-value of 0.017.

However, the interaction term between gender and olanzapine showed a borderline significance (B_7_= -82.61× 10^3^/µL, *95% CI*: -179.29 – 14.08, *p* = 0.092).

The interaction between olanzapine intake, the EDDO, and gender demonstrated a statistically significant influence on the re-lymph count (*p* = 0.004) and was analyzed in depth using estimated marginal means (EMMs). Among females with an EDDO of 19.6 [mg], the mean of the relative lymphocyte count was significantly lower in those using olanzapine (59.9 ×10^6^/µL, *95% CI*: 30.56 – 89.3) compared to those not using olanzapine (112.1 ×10^6^/µL, *95% CI*: 87.71 – 136.4). The difference between the two groups was statistically significant (estimate = 52.1 ×10^6^/µL, *p* = 0.008), indicating that the use of olanzapine was associated with a lower re-lymph count among females with an EDDO of 19.6 [mg].

Among males with an EDDO of 19.6 [mg], the mean Re-Lymph count was slightly higher in those using olanzapine (62.6 ×10^6^/µL, *95% CI*: 41.17 – 84.0) compared to those not using olanzapine (47.4 ×10^6^/µL, *95% CI:* 6.66 – 88.2). However, this difference was not statistically significant (estimate= -15.1 ×10^6^/µL, *p* = 0.507), suggesting that the use of olanzapine did not have a significant effect on the WBC count in males with an EDDO of 19.6 [mg].

| *Predictors* | **Re-Lymph [×10^6^/µL]** | | |
| --- | --- | --- | --- |
|  | *Estimates* | *CI 95%* | *p* |
| (Intercept) | 23.27 | -26.30 – 72.84 | 0.349 |
| age | 1.24 | -0.23 – 2.71 | 0.096 |
| EDDO [mg] | 2.78 | 1.59 – 3.97 | **<0.001** |
| gender [male] | 55.08 | -3.06 – 113.21 | 0.063 |
| olanzapine [yes] | 14.28 | -51.74 – 80.30 | 0.665 |
| EDDO × gender [male] | -6.11 | -9.84 – -2.38 | **0.002** |
| EDDO × olanzapine [yes] | -3.39 | -6.15 – -0.63 | **0.017** |
| gender [male] × olanzapine [yes] | -82.61 | -179.29 – 14.08 | 0.092 |
| EDDO × gender[male] × olanzapine [yes] | 7.65 | 2.62 – 12.68 | **0.004** |

Supplementary Table S11. Krzyściak Wirginia, Title: Statistical Predictive Model of Schizophrenia. Results of fitting regression model with Re-Lymph as outcome, n_obs_ = 51

### RBC [×10^6^/µL]

In this study, we evaluated the effects of various predictors, including age, EDDO, gender, and olanzapine intake, on RBC count. Our regression model, based on 51 observations, accounted for approximately 32.6% of the variance in RBC counts (*R^2^* = 0.326), with an adjusted *R^2^*_adj_ of 0.198 after factoring in the number of predictors. The estimates, along with their 95% CIs and associated *p*-values, were reported in Supplementary Table S12.

Our findings suggest that age, EDDO, gender, and olanzapine intake, as well as their interactions, did not significantly influence RBC counts in study sample. This indicated that these factors may not be relevant predictors of RBC count in this context.

Detailed interpretation:

Age showed no effect (B_1_ = 0.00×10^6^/µL, *95% CI*: -0.01 – 0.02, *p* = 0.953), as did EDDO (estimate: B_2_ = -0.00×10^6^/µL, *95% CI*: -0.01 – 0.01, *p* = 0.852) and olanzapine intake (B_4_ = 0.01×10^6^/µL, *95% CI*: -0.66 – 0.68, *p* = 0.975). The effect of gender, as indicated by being male, was not significant either (B_3_ = 0.49×10^6^/µL, *95% CI*: -0.10 – 1.08, *p* = 0.102).

Furthermore, the interaction terms between EDDO and gender, EDDO and olanzapine, and gender and olanzapine showed no significant effect on the RBC count. Similarly, the three-way interaction term between EDDO, gender, and olanzapine was not significant.

| *Predictors* | **RBC [×10^6^/µL]** | | |
| --- | --- | --- | --- |
|  | *Estimates* | *CI 95%* | *p* |
| (Intercept) | 4.68 | 4.18 – 5.19 | **<0.001** |
| age | 0.00 | -0.01 – 0.02 | 0.953 |
| EDDO [mg] | -0.00 | -0.01 – 0.01 | 0.852 |
| gender [male] | 0.49 | -0.10 – 1.08 | 0.102 |
| olanzapine [yes] | 0.01 | -0.66 – 0.68 | 0.975 |
| EDDO × gender [male] | 0.00 | -0.04 – 0.04 | 0.995 |
| EDDO × olanzapine [yes] | -0.00 | -0.03 – 0.03 | 0.899 |
| gender [male] × olanzapine [yes] | -0.12 | -1.09 – 0.86 | 0.813 |
| EDDO × gender[male] × olanzapine [yes] | 0.01 | -0.04 – 0.06 | 0.770 |

Supplementary Table S12. Krzyściak Wirginia, Title: Statistical Predictive Model of Schizophrenia. Results of fitting regression model with RBC as outcome.

### Taurine/Cr+PCr ×10^3^ (rear rim turn TE 30ms)

This study investigated the influence of several predictors, including age, the EDDO, gender, and olanzapine intake, on Taurine Cr PCr (measured at the rear rim turn TE 30ms). Our regression model, based on 50 observations, accounted for approximately 32.0% of the variance in Taurine/Cr+PCr values (*R^2^*= 0.320), with an adjusted *R^2^*_adj_ = 0.187 after accounting for the number of predictors. The estimates, along with their 95% CIs and associated *p*-values, were reported in Supplementary Table S13.

Age significantly influenced Taurine/Cr+PCr values, with older age associated with lower levels. Other factors such as EDDO, gender, and olanzapine intake, as well as their interactions, did not demonstrate a significant effect.

Detailed interpretation:

Age was the only predictor to show a significant impact on Taurine Cr PCr, with an estimate of B_1_ = -1.86 × 10^3^ (*95% CI*: -3.28 – -0.44, *p* = 0.012), indicating that Taurine/Cr+PCr decreased with increasing age. EDDO (B_2_ = 0.07 × 10^3^, *95% CI*: -1.06 – 1.19, *p* = 0.905), gender, as indicated by being male (B_3_ = 22.53 × 10^3^, *95% CI*: -32.80 – 77.86, *p* = 0.416), and olanzapine intake (B_4_ = -50.55 × 10^3^, *95% CI*: -113.85 – 12.75, *p* = 0.114) did not significantly affect Taurine/Cr+PCr.

The interaction terms between EDDO and gender (estimate: -3.11, 95% CI: -6.62 – 0.40, *p* = 0.081) and EDDO and olanzapine (B5 = 2.37× 10^3^, *95% CI*: -0.23 – 4.97, *p* = 0.072) showed borderline significance. The interaction terms between gender and olanzapine, and the three-way interaction term between EDDO, gender, and olanzapine, did not significantly influence Taurine/Cr+PCr.

| *Predictors* | **Taurine/Cr+PCr ×10^3^**  (rear rim turn TE 30ms) | | |
| --- | --- | --- | --- |
|  | *Estimates* | *CI 95%* | *p* |
| (Intercept) | 96.85 | 46.77 – 146.92 | **<0.001** |
| age | -1.86 | -3.28 – -0.44 | **0.012** |
| EDDO [mg] | 0.07 | -1.06 – 1.19 | 0.905 |
| gender [male] | 22.53 | -32.80 – 77.86 | 0.416 |
| olanzapine [yes] | -50.55 | -113.85 – 12.75 | 0.114 |
| EDDO × gender [male] | -3.11 | -6.62 – 0.40 | 0.081 |
| EDDO × olanzapine [yes] | 2.37 | -0.23 – 4.97 | 0.072 |
| gender [male] × olanzapine [yes] | 13.85 | -78.17 – 105.87 | 0.763 |
| EDDO × gender[male] × olanzapine [yes] | 0.74 | -3.99 – 5.48 | 0.753 |

Supplementary Table S13. Krzyściak Wirginia, Title: Statistical Predictive Model of Schizophrenia. Results of fitting regression model with Taurine Cr PCr as outcome.

### Cr+PCr conc. ×10^6^ (rear rim turn TE 30ms)

The study aimed to investigate the effect of various predictors, including age, the EDDO, gender, and olanzapine intake, on R 30 Cr+PCr concentration. The regression model utilized 50 observations and explained roughly 35.8% of the variance in R 30 Cr+PCr concentrations (*R^2^* = 0.358), with an adjusted *R^2^*_adj_ = 0.233 after considering the number of predictors. The estimates, along with their 95% CIs and associated *p*-values, were reported in Supplementary Table S14.

The study found that EDDO and olanzapine intake significantly influence Cr PCr concentrations, with both associated with lower concentrations. Importantly, the interaction between EDDO and olanzapine intake was also found to be significant. This suggested that the relationship between EDDO and Cr+PCr concentrations varied depending on whether olanzapine is being taken. Specifically, it appeared that the negative effect of EDDO on Cr +PCr concentrations was more pronounced among patients with olanzapine intake.

Detailed interpretation:

The intercept was significant (*B_0_* = 161.95×10^6^, *95% CI*: 143.10 – 180.81, *p* < 0.001), suggesting a baseline concentration of Cr PCr in the case of an individual aged zero with EDDO = 0 mg, not taking olanzapine, female. Age was not a significant predictor (*B_1_*= -0.30×10^6^, *95% CI*: -0.84 – 0.23, *p* = 0.263), and neither was gender, as indicated by being male (*B_3_*= -7.44×10^6^, *95% CI*: -28.27 – 13.39, *p* = 0.475). The EDDO showed a significant negative effect (*B_2_* = -0.46×10^6^, *95% CI*: -0.88 – -0.04, *p* = 0.034), suggesting that higher EDDO was associated with lower Cr PCr concentrations. Olanzapine intake also had a significant negative effect (*B_4_* =-24.57×10^6^, *95% CI*: -48.40 – -0.74, *p* = 0.044), indicating lower Cr PCr concentrations in those taking olanzapine.

The interaction term between EDDO and olanzapine was also significant (*B_6_* = 1.43, *95% CI*: 0.45 – 2.41, *p* = 0.005), suggesting that the effect of EDDO on R 30 Cr P Cr concentrations was higher in those taking olanzapine compared to those not taking it. The interaction terms between EDDO and gender, gender and olanzapine, and the three-way interaction term between EDDO, gender, and olanzapine were not significant.

| *Predictors* | **Cr+PCr conc. ×10^6^**  (rear rim turn TE 30ms) | | |
| --- | --- | --- | --- |
|  | *Estimates* | *CI 95%* | *p* |
| (Intercept) | 161.95 | 143.10 – 180.81 | **<0.001** |
| age | -0.30 | -0.84 – 0.23 | 0.263 |
| EDDO [mg] | -0.46 | -0.88 – -0.04 | **0.034** |
| gender [male] | -7.44 | -28.27 – 13.39 | 0.475 |
| olanzapine [yes] | -24.57 | -48.40 – -0.74 | **0.044** |
| EDDO × gender [male] | -0.48 | -1.80 – 0.84 | 0.465 |
| EDDO × olanzapine [yes] | 1.43 | 0.45 – 2.41 | **0.005** |
| gender [male] × olanzapine [yes] | 7.70 | -26.94 – 42.35 | 0.656 |
| EDDO × gender[male] × olanzapine [yes] | -0.13 | -1.92 – 1.65 | 0.882 |

Supplementary Table S14. Krzyściak Wirginia, Title: Statistical Predictive Model of Schizophrenia. Results of fitting regression model with Cr+PCr conc. as outcome, n_obs_ = 50

Insulin [µU/mL]

The study aimed to evaluate the impact of various predictors, including age, EDDO, gender, and olanzapine intake, on insulin levels. The multiple regression model, based on 51 observations, explained approximately 18.2% of the variance in insulin levels (*R²* = 0.182). The estimates, along with their 95% CIs and associated *p*-values, were reported in Supplementary Table S15.

This study did not find any significant associations between the predictors examined (age, EDDO, gender, olanzapine intake) and insulin levels. Furthermore, no significant interactions between these predictors were observed. Given the low adjusted R², other unexamined factors may be contributing to the variance in insulin levels.

Detailed interpretation:

None of the predictors showed a statistically significant impact on insulin levels. Specifically, age (*B_1_*= 0.15 µU/mL, *95% CI*: -0.28 – 0.58, *p* = 0.490), EDDO (*B_2_*= 0.11 µU/mL, *95% CI*: -0.23 – 0.46, *p* = 0.511), gender, as indicated by being male (*B_3_* = 4.61 µU/mL, *95% CI*: -12.32 – 21.54, *p* = 0.586), and olanzapine intake (*B_4_ =*13.46 µU/mL, *95% CI*: -5.77 – 32.68, *p* = 0.165) did not individually have significant effects on insulin levels.

Similarly, none of the interaction terms were statistically significant. This includes the interactions between EDDO and gender (B_5_ = -0.06 µU/mL, *95% CI*: -1.15 – 1.02, *p* = 0.908), EDDO and olanzapine (*B_6_* = -0.11 µU/mL, *95% CI*: -0.91 – 0.70, *p* = 0.789), gender and olanzapine (*B_7_* = -25.17 µU/mL, *95% CI*: -53.32 – 2.99, *p* = 0.078), and the three-way interaction between EDDO, gender, and olanzapine (*B_8_* = 0.34 µU/mL, *95% CI*: -1.12 – 1.81, *p* = 0.639).

| *Predictors* | **Insulin [µU/mL]** | | |
| --- | --- | --- | --- |
|  | *Estimates* | *CI 95%* | *p* |
| (Intercept) | 7.48 | -6.95 – 21.91 | 0.302 |
| age | 0.15 | -0.28 – 0.58 | 0.490 |
| EDDO [mg] | 0.11 | -0.23 – 0.46 | 0.511 |
| gender [male] | 4.61 | -12.32 – 21.54 | 0.586 |
| olanzapine [yes] | 13.46 | -5.77 – 32.68 | 0.165 |
| EDDO × gender [male] | -0.06 | -1.15 – 1.02 | 0.908 |
| EDDO × olanzapine [yes] | -0.11 | -0.91 – 0.70 | 0.789 |
| gender [male] × olanzapine [yes] | -25.17 | -53.32 – 2.99 | 0.078 |
| EDDO × gender[male] × olanzapine [yes] | 0.34 | -1.12 – 1.81 | 0.639 |

Supplementary Table S15. Krzyściak Wirginia, Title: Statistical Predictive Model of Schizophrenia. Results of fitting regression model with Insulin [µU/mL] as outcome, n_obs_ = 51.

### Glucose [mmol/l]

This study aimed to understand the influence of several predictors—including age, EDDO, gender, and olanzapine intake—on glucose levels (measured in mmol/l). The multiple regression model used 51 observations and explained approximately 16.1% of the variance in glucose levels (*R*² = 0.161), The estimates, along with their 95% CIs and associated p-values, were reported in Supplementary Table S16.

This study did not find any significant associations between the predictors examined (age, EDDO, gender, olanzapine intake) and glucose levels. Moreover, no significant interactions between these predictors were observed. Given the low adjusted R², other unexamined factors may be contributing to the variance in glucose levels.

Detailed interpretation:

The intercept was significant (B_0_ = 3.81 mmol/l, *95% CI*: 2.38 – 5.25, *p* < 0.001), indicating the baseline glucose level in the case of an individual aged zero with EDDO = 0 mg, not taking olanzapine, female. However, none of the predictors showed a statistically significant impact on glucose levels. Specifically, age (*B_1_* = 0.03 mmol/l, *95% CI*: -0.01 – 0.07, *p* = 0.194), EDDO (*B_2_* = 0.01 mmol/l, *95% CI*: -0.03 – 0.04, *p* = 0.719), gender, as indicated by being male (*B_3_* =0.54 mmol/l, *95% CI*: -1.14 – 2.22, *p* = 0.520), and olanzapine intake (*B_4_* = 0.72 mmol/l, *95% CI*: -1.19 – 2.63, *p* = 0.451) did not individually have significant effects on glucose levels.

Furthermore, none of the interaction terms were statistically significant. This includes the interactions between EDDO and gender (*B_5_*= 0.03 mmol/l, 95% CI: -0.07 – 0.14, *p* = 0.527), EDDO and olanzapine (*B_6_* = -0.01 mmol/l, *95% CI*: -0.09 – 0.07, *p* = 0.777), gender and olanzapine (*B_7_* = -1.52 mmol/l, *95% CI*: -4.31 – 1.28, *p* = 0.281), and the three-way interaction between EDDO, gender, and olanzapine (*B_8_* = -0.00 mmol/l, *95% CI*: -0.15 – 0.14, *p* = 0.961).

| *Predictors* | **Glucose [mmol/l]** | | |
| --- | --- | --- | --- |
|  | *Estimates* | *CI 95%* | *p* |
| (Intercept) | 3.81 | 2.38 – 5.25 | **<0.001** |
| age | 0.03 | -0.01 – 0.07 | 0.194 |
| EDDO [mg] | 0.01 | -0.03 – 0.04 | 0.719 |
| gender [male] | 0.54 | -1.14 – 2.22 | 0.520 |
| olanzapine [yes] | 0.72 | -1.19 – 2.63 | 0.451 |
| EDDO × gender [male] | 0.03 | -0.07 – 0.14 | 0.527 |
| EDDO × olanzapine [yes] | -0.01 | -0.09 – 0.07 | 0.777 |
| gender [male] × olanzapine [yes] | -1.52 | -4.31 – 1.28 | 0.281 |
| EDDO × gender[male] × olanzapine [yes] | -0.00 | -0.15 – 0.14 | 0.961 |

Supplementary Table S16. Krzyściak Wirginia, Title: Statistical Predictive Model of Schizophrenia. Results of fitting regression model with Glucose [mmol/l] as outcome, n_obs_ = 51.

### BDI II total score

This study aimed to evaluate the impact of various predictors, including age, the effective dose of all drugs per olanzapine (EDDO), gender, and olanzapine intake, on the total score of the Beck Depression Inventory (BDI II). The multiple regression model, derived from 46 observations, accounted for approximately 14.3% of the variance in BDI II scores (R² = 0.143). The estimates, along with their 95% CIs and associated p-values, were reported in Supplementary Table S17.

This study did not find significant associations between the predictors examined (age, EDDO, gender, olanzapine intake) and BDI II scores. Furthermore, no significant interactions between these predictors were observed. It is likely that other unexamined factors may significantly contribute to the variance in BDI II scores.

Detailed interpretation:

None of the predictors or their interactions were statistically significant. Specifically, age (*B_1_* = 0.03, *95% CI*: -0.55 – 0.61, *p* = 0.926), EDDO (*B_2_* = 0.33, *95% CI*: -0.10 – 0.77, *p* = 0.129), gender, as indicated by being male (*B_3_* = 9.57, *95% CI*: -13.62 – 32.76, *p* = 0.408), and olanzapine intake (*B_4_* = 16.87, *95% CI*: -10.31 – 44.05, *p* = 0.216) did not individually have significant effects on BDI II scores.

Similarly, the interaction terms between EDDO and gender (*B_5_* = -0.58, *95% CI*: -2.10 – 0.95, p = 0.447), EDDO and olanzapine (*B_6_* = -0.23, *95% CI*: -1.33 – 0.87, p = 0.669), gender and olanzapine (*B_7_* = -14.33, *95% CI*: -53.52 – 24.86, *p* = 0.464), and the three-way interaction between EDDO, gender, and olanzapine (*B_8_* = 0.38, *95% CI*: -1.65 – 2.40, *p* = 0.709) were also not statistically significant.

| *Predictors* | **BDI II total score** | | |
| --- | --- | --- | --- |
|  | *Estimates* | *CI 95%* | *p* |
| (Intercept) | 7.96 | -10.72 – 26.63 | 0.393 |
| age | 0.03 | -0.55 – 0.61 | 0.926 |
| EDDO [mg] | 0.33 | -0.10 – 0.77 | 0.129 |
| gender [male] | 9.57 | -13.62 – 32.76 | 0.408 |
| olanzapine [yes] | 16.87 | -10.31 – 44.05 | 0.216 |
| EDDO × gender [male] | -0.58 | -2.10 – 0.95 | 0.447 |
| EDDO × olanzapine [yes] | -0.23 | -1.33 – 0.87 | 0.669 |
| gender [male] × olanzapine [yes] | -14.33 | -53.52 – 24.86 | 0.464 |
| EDDO × gender[male] × olanzapine [yes] | 0.38 | -1.65 – 2.40 | 0.709 |

Supplementary Table S17. Krzyściak Wirginia, Title: Statistical Predictive Model of Schizophrenia. Results of fitting regression model with BDI II total score as outcome, n_obs_ = 46.

**4. Methods**

*4.1. Participants*

The study's exclusion criteria were comprehensive, covering various aspects. Participants under court-ordered treatment, those with limited legal capacity, and individuals with intellectual disabilities were excluded. Additionally, the study did not include individuals with severe cardiovascular diseases, diabetes, insulin resistance, metabolic syndrome, or a history of central nervous system disorders. The study imposed restrictions on individuals taking specific medications, such as clozapine, treatment within the last 3 months before the study, changes or modifications to antipsychotic treatment within 12 weeks before the study, or recent use (within 3 days before the study) of antibiotics, non-steroidal anti-inflammatory drugs, corticosteroids, vitamin supplements, antioxidants, probiotics, psychoactive, or narcotic substances. Furthermore, participants with a diagnosis of substance dependence according to ICD-10 or individuals with a recent history of alcohol or substance abuse (excluding tobacco) within 3 months before the study were ineligible. The exclusion criteria also encompassed intense affective symptoms, hyperactivity, or psychomotor agitation, pregnancy, breastfeeding, severe claustrophobia, inability to remain in a supine position (due to spinal deformity), the presence of pacemakers, drug delivery pumps, cochlear implants, neurostimulators, or other implanted electronic devices, artificial heart valves, vascular clips, and metallic orthopedic implants such as artificial joints, wires, screws, and stabilizers. Additionally, metallic foreign bodies like iron filings or other metal instrumentation contraindicated in magnetic resonance imaging (MRI) and/or magnetic resonance spectroscopy (MRS) were grounds for exclusion. Lastly, the age criteria for exclusion encompassed individuals aged ≤13 and ≥40 years or those without a diagnosis by a psychiatrist according to ICD-10.

*4.3. Magnetic Resonance Techniques*

Magnetic resonance imaging (MRI) and spectroscopy (MRS) were performed by the 3 T Siemens Magnetom Vida Fit whole-body magnetic resonance scanner. The MR system used a 20-channel head coil and strong whole-body gradients (amplitude of 45 mT/m, rise rate of 200 T/m/s) enabling accurate and fast imaging. MRI and MRS examinations were performed on the same magnetic resonance scanner and on the same day. In the first step, each participant underwent brain imaging according to the standard MRI protocol to exclude possible morphological changes in the central nervous system. The non-contrast brain scanning consisted of the following sequences:

1. 3D T2 sequences in the sagittal plane (scanning sequences: spin echo, space; slice thickness 1.0 mm; spacing 0.9 mm; Repetition Time (TR) 3200 ms; Time to Echo (TE) 260 ms; Flip Angle (FA) 120°; Field of View (FOV) 25 cm and matrix 256 × 256 pixels).

2. 3D T1 sequences in the sagittal plane (scanning sequences: gradient, mprage; slice thickness 1.2 mm; spacing 0.9 mm; TR 2300 ms; TE 260 ms; FA 9°; FOV 25 cm and matrix 256 × 248 pixels).

3. 3D dark fluid sequences in the sagittal plane (scanning sequences: spin echo resolve; slice thickness 1.0 mm; spacing 0.9 mm; TR 7000 ms; TE 394 ms; Inversion Time (TI) 2050 ms; FA 120°; FOV 25 cm and matrix 256 × 288 pixels).

4. 2D DWI sequences in the axial plane (scanning sequences: epi; slice thickness 3.0 mm; spacing 3.9 mm; TR 4120 ms; TE 65 ms; b 1000 s/mm²; FA 160°; FOV 23 cm and matrix 448 × 448 pixels).

T1, T2, and dark fluid images were automatically reconstructed into coronal and axial planes with a 1.00 mm slice thickness and 0 mm spacing. Three-dimensional T2 imaging was conducted to visualize anatomical brain structures and plan the volume of interest (VOI) position for spectroscopy. The brain imaging analysis was performed by a radiologist with over 20 years of experience. The next step involved MRS, which was carried out using the single-voxel spectroscopy technique (SVS). The acquisition parameters for MRS included TE values of 30 ms and 144 ms, TR of 2000 ms, and 64 averages acquired. In the study, two echo times (TE) were selected to record metabolites with both long and short relaxation times. MRS signals were obtained from two locations situated symmetrically in the anterior cingulate cortex (ACC) and posterior cingulate cortex (PCC) (Supplementary Figure S1). The VOI was adapted to the anatomical size of the location from which the spectrum was obtained, with a mean VOI volume of 3.4 cm³. The VOI was positioned away from any source of susceptibility artifacts. MRS data were analyzed using LCModel [1], a tool dedicated to the automatic quantitation of in vivo proton MR spectra, and concentrations of metabolites were measured based on their resonance areas.


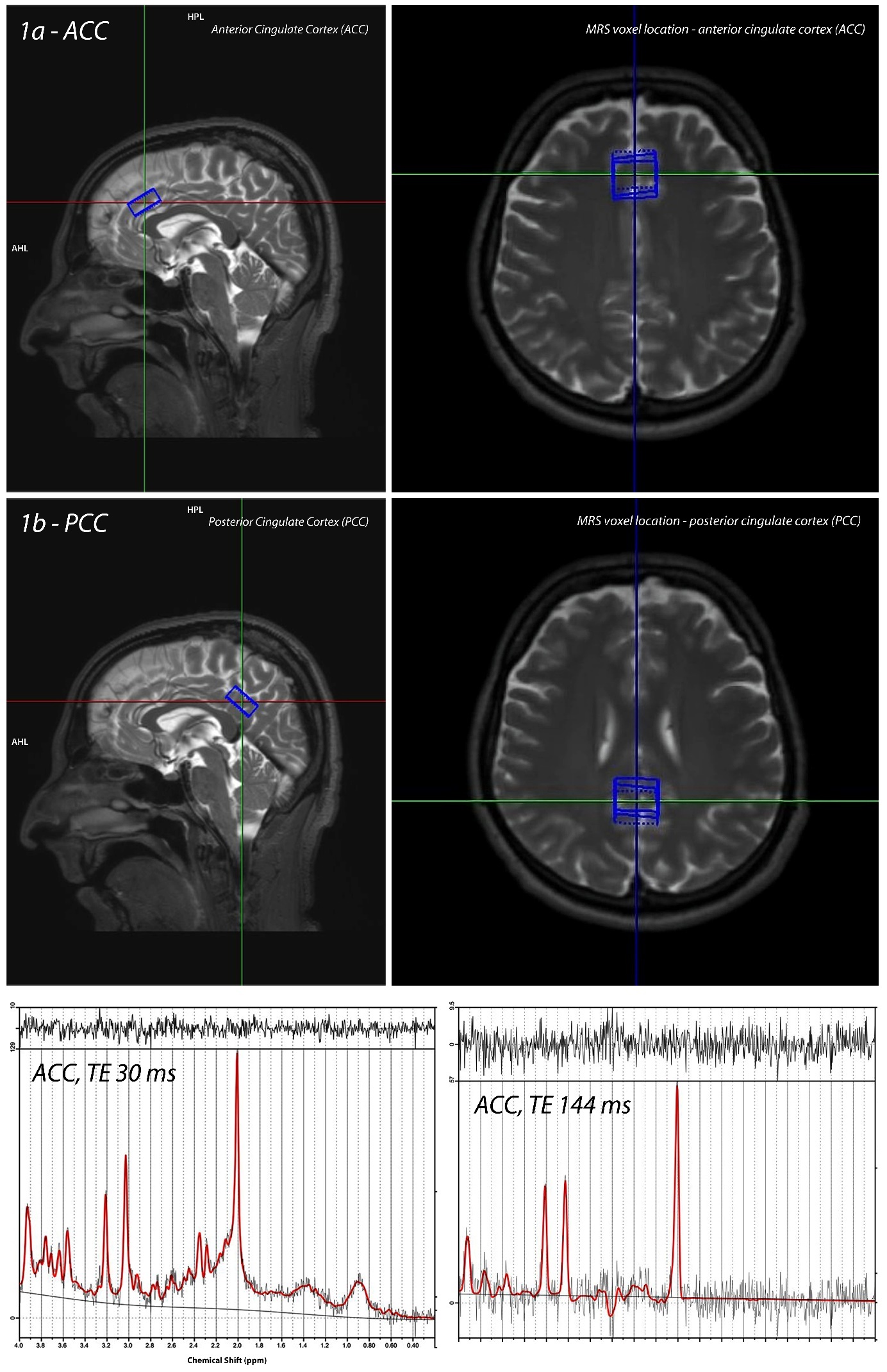


Supplementary Figure S1. Krzyściak Wirginia, Title: Statistical Predictive Model of Schizophrenia. a. MRS voxel location—anterior cingulate cortex (ACC), b. MRS voxel location—posterior cingulate cortex (PCC). MRS spectrum with ACC for Time to Echo (TE) 30 ms and 144 ms. Metabolite spectrum in the anterior cingulate cortex (ACC) in schizophrenia patients at 3 T. L-Alanine (Ala) at 1.48 ppm, Aspartate (Asp) at 3.8 ppm, Creatine (Cr) at 3.02 ppm, Creatine/Phosphocreatine (Cr/PCr) at 3.02 ppm, γ-aminobutyric acid (GABA) at 2.3 ppm, Glucose (Glc) at 3.43 ppm, Glutamine (Gln) at 2.45 and 3.7 ppm, Glutamate (Glu) at 2.1 and 3.7 ppm, Glutathione (GSH) at 3.7 ppm, myo-Inositol (Ins) at 3.6 ppm, L-Lactate (Lac) at 1.33 ppm, N-Acetylaspartate (NAA) at 2.02 ppm, Taurine (Tau) at 3.42 ppm.

*4.4. Statistical Analysis*

For this analysis, a statistical significance level was established at α = 0.05. Numeric variables that exhibited non-normal distributions were denoted as *Mdn* (*Q1*, *Q3*), representing the median and interquartile range. Categorical variables were quantified as counts (*n*) and proportions (%).

The dependent variables, denoted as *Yi*, adhered to a binomial distribution. The model's coefficients β for *k* predictors, represented were determined via the maximum likelihood estimation method. The parameter βj in the model was interpreted as the incremental effect that a single unit alteration in the *j*-th predictor has on the odds ratio, as outlined by equation (3). This meant that for each one-unit increase in the predictor variable, the odds ratio changed by a factor of βj, holding all other variables constant. In the framework of this analysis, *A* and *B* served as identifiers for the study groups. *P* corresponded to the probability of schizophrenia occurrence within each respective group, while *S* indicated the likelihood of this occurrence probability, following the model proposed by Danieluk (2010) [2]. The description and modeling of the error (residuals) distribution and the joint function were based on the Gaussian distribution. In this model, the residuals, also known as errors, were assumed to follow a normal distribution. Additionally, equation (2) was adapted using a probit link function, in which the probability of schizophrenia occurrence was encapsulated by equation (4), with Φ symbolizing the standard cumulative normal distribution function. The choice of the most suitable link function was made by assessing the values of the information criteria, specifically Akaike's Information Criterion (AIC) and the Bayesian Information Criterion (BIC). The efficacy of the fitted model was evaluated by verifying the adherence to the essential assumptions of the logistic regression model. Multicollinearity among the model terms was scrutinized by calculating the Variance Inflation Factor (VIF) [3]. A VIF value less than 3.0 indicated a low level of correlation between that specific predictor and the remaining predictors [4], suggesting that the model was not overly influenced by redundant information. The normality of the distribution of the residuals of the model was checked using the Kolmogorov-Smirnov normality test. The model's predictive capabilities were put to the test using real-world data, adhering to the methodology proposed by Statisticat LLC [5] and drawing upon the works of Gelman and Hill (2007) [6] as well as Gelman et al. (2013) [7]. The discerning capacity of the fitted model was evaluated by constructing the Receiver Operating Characteristic (ROC) curve and computing the Area Under the Curve (AUC). The goodness of fit for the regression model was evaluated using both the Hosmer-Lemeshow test [8] and the Osius and Rojek test [9]. The analysis of the average marginal effect (the mean of unit-specific partial derivatives across the sample) was conducted on a linear predictor scale using the "link" type, as detailed by Greene (2012) [10]. The determination and evaluation of optimal cutpoints between the control and test groups were carried out using the method that maximizes the sum of sensitivity (Se) and specificity (Sp), with a "more or equal" direction. A bootstrapping procedure, a resampling technique used to estimate statistics on a population by sampling a dataset with replacement, was employed for this purpose. The variability and out-of-sample performance were assessed based on 1000 bootstrap samples. These samples were of the same size as the original dataset and were randomly drawn from it with replacement. The cutpoint estimation was performed for the in-bag sample (the observations included in the bootstrap sample), and the determined cutpoint was applied to both the in-bag and out-of-bag observations (those not included in the bootstrap sample) [11]. The analyses were conducted using the R statistical language (version 4.1.10.) [12] in conjunction with the following packages: *gtsummary* (version 1.6.2.) [13], providing table-making tools for summarizing data and analysis results, *MASS* (version 7.3.57.) [14], supplying functions for applied statistics, *sjPlot* (version 2.8.14.) [15], for data visualization for statistics in social science, *performance* (version 0.10.4.) [16], for computing metrics to assess model quality, *cutpointr* (version 1.1.2.) [11], for optimal cutpoint estimation in diagnostic tests, *margins* (version 0.3.26.) [17], for computing marginal effects at the mean or average marginal effects, *DHARMa* (version 0.4.6.) [18], that provides residual diagnostics for regression models, *report* (version 0.5.7.) [19], that facilitates the automatic generation of documents and reporting of results, *readxl* (version 1.3.1.) [20] and *dplyr* (version 1.1.2.) [21], for i/o operations and data manipulation.

Equation (2), Krzyściak Wirginia, Title: Statistical Predictive Model from Periphery Immunity to Central Domain Through Clinical Interview in the Evaluation of Schizophrenia Predictors: New Directions.

$$logit(p_{i})=ln(\frac{p_{i}}{1-p_{i}})={\beta0+\beta}_{1}\cdot x_{1,i}+...+\beta_{k}\cdot x_{k,i}(1)$$

Equation (3), Krzyściak Wirginia, Title: Statistical Predictive Model from Periphery Immunity to Central Domain Through Clinical Interview in the Evaluation of Schizophrenia Predictors: New Directions.

$${OR}_{AxB}=\frac{S(A)}{S(B)}=\frac{\frac{P(A)}{1-P(A)}}{\frac{P(B)}{1-P(B)}} = \frac{P(A) x (1-P(B))}{P(B) x (1-P(A))} (2)$$

Equation (4), Krzyściak Wirginia, Title: Statistical Predictive Model from Periphery Immunity to Central Domain Through Clinical Interview in the Evaluation of Schizophrenia Predictors: New Directions.

$$P\left( Y=1 | x_{1i},\ldots, x_{1k} \right)=Ф\left( \beta_{0}+\beta_{1}x_{1i},+\ldots+ {\beta_{k}x}_{1k} \right), (3)$$

**References**

1. Provencher, S. W. Automatic quantitation of localized in vivo 1H spectra with LCModel. *NMR Biomed* **14**, 260–264 (2001).

2. Danieluk, B. Zastosowanie regresji logistycznej w badaniach eksperymentalnych. *Psychologia Społeczna* **2–3**, 199–216 (2010).

3. James, G., Witten, D., Hastie, T. & Tibshirani, R. *An introduction to statistical learning: with applications in R*. (Springer, 2013). doi:10.1007/978-1-4614-7138-7_1.

4. Zuur, A. F., Ieno, E. N. & Elphick, C. S. A protocol for data exploration to avoid common statistical problems. *Methods Ecol Evol* **1**, 3–14 (2010).

5. Statisticat LLC. Laplaces Demon: complete environment for Bayesian Inference (2013).

6. Gelman, A. & Hill, J. *Data analysis using regression and multilevel/hierarchical models*. *Data Analysis Using Regression and Multilevel/Hierarchical Models* (Cambridge University Press, 2006). doi:10.1017/CBO9780511790942.

7. Gelman, A. *et al.* *Bayesian Data Analysis*. (Chapman and Hall/CRC, 2013). doi:https://doi.org/10.1201/b16018.

8. Hosmer, D. W., Hosmer, T., Le Cessie, S. & Lemeshow, S. A comparison of goodness-of-fit tests for the logistic regression model. *Stat Med* **16**, 965–980 (1997).

9. Osius, G. & Rojek, D. Normal goodness-of-fit tests for multinomial models with large degrees of freedom. *J Am Stat Assoc* **87**, 1145–1152 (1992).

10. Greene, W. H. *Econometric analysis* (Pearson, 2012).

11. Thiele, C. & Hirschfeld, G. cutpointr: Improved estimation and validation of optimal cutpoints in R. *J Stat Softw* **98**, 1–27 (2021).

12. R Core Team. R: A language and environment for statistical computing. R foundation for statistical computing, Vienna, Austria (2021).

13. Sjoberg, D. D., Whiting, K., Curry, M., Lavery, J. A. & Larmarange, J. Reproducible summary tables with the summary package. *R Journal* **13**, 570–580 (2021).

14. Venables, W. N. & Ripley, B. D. *Modern applied statistics with S, fourth edition*. (Springer, 2002). doi:10.1007/978-0-387-21706-2.

15. Lüdecke, D. sjPlot: Data visualization for statistics in social science. R package version 2.8.14. (2023).

16. Lüdecke, D., Ben-Shachar, M. S., Patil, I., Waggoner, P. & Makowski, D. performance: An R package for assessment, comparison and testing of statistical models. *J Open Source Softw* **6**, (2021).

17. Leeper, T. margins: Marginal effects for model objects. R package version 0.3.26. (2021).

18. Hartig, F. DHARMa: Residual diagnostics for hierarchical (multi-level / mixed) regression models. R package version 0.4.6. (2022).

19. Makowski, D. *et al.* Automated results reporting as a practical tool to improve reproducibility and methodological best practices adoption (2023).

20. Wickham, H. & Bryan, J. readxl: Read excel files. R package version 1.3.1. (2019).

21. Wickham, H., François, R., Henry, L., Müller, K. & Vaughan, D. dplyr: A grammar of data manipulation. R package version 1.1.2. (2023).
